# Heterogeneity‐Guided Interlayer Tuning in Vermiculite‐MOF Membranes for Efficient Li^+^/Mg^2+^ Separation

**DOI:** 10.1002/advs.74597

**Published:** 2026-02-27

**Authors:** Liheng Dai, Kecheng Guan, Mengyang Hu, Erda Deng, Pengfei Zhang, Zhan Li, Yongxuan Shi, Xiao Xu, Xueru Yan, Hideto Matsuyama

**Affiliations:** ^1^ Research Center for Membrane and Film Technology Kobe University Kobe Japan; ^2^ Department of Chemical Science and Engineering Kobe University Kobe Japan

**Keywords:** 2D membrane, lithium extraction, MOF nanosheets, vermiculite

## Abstract

Precise separation of Li^+^/Mg^2+^ with similar ionic size remains a critical challenge for two‐dimensional (2D) membranes, largely due to the inherently tortuous and poorly regulated transport channels in the 2D interlayer. Herein, we propose a heterogeneity‐guided interlayer engineering strategy to reprogram the lamellar ion transport pathways of vermiculite (Vm) membranes through the incorporation of ultrathin ZrBTB (Zr) metal organic framework nanosheets. Owing to the intrinsic electrostatic environment and sub‐nanometer apertures, the resulting vermiculite/ZrBTB heterolamina membranes exhibit reorganized interlayer nanochannels without changing the average interlayer spacing. Systematic structural analysis reveals that ZrBTB incorporation suppresses long‐range lamellar ordering while enriching sub‐nanoconfinement domains and partially reversing the channel‐wall charge. These synergistic modifications generate enhanced Li^+^ transport pathways and a strengthened electrostatic/steric exclusion environment for Mg^2+^. As a result, the optimum membrane achieves a Li^+^ permeation rate of 0.379 mol m^−2^ h^−1^ and an outstanding Li^+^/Mg^2+^ selectivity of ∼46.2, surpassing most diffusion‐based separation membranes. Comparative studies using CuTCPP nanosheets further confirm that channel size, charge, and energy barriers can be effectively tuned through rational selection of 2D MOF building blocks. This work establishes interlayer heterogeneity as a powerful and generalizable design parameter for constructing next‐generation 2D membranes for efficient ion sieving under mild conditions.

## Graphical abstract

Heterogeneity‐guided interlayer engineering is achieved by integrating porous MOF nanosheets into a 2D vermiculite laminate, reprogramming ion transport pathways. The resulting hybrid membranes combine sub‐nanoconfinement and charge heterogeneity, enabling accelerated Li^+^ transport and reinforced Mg^2+^ exclusion. This mechanism‐driven design based on interlayer confinement and charge environment enables precise Li^+^/Mg^2+^ separation beyond conventional laminar membrane limitations under mild diffusion‐driven conditions.

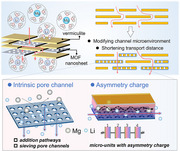

## 1 Introduction

Laminar materials with ultrahigh lateral‐to‐thickness ratios and well‐defined interlayer nanochannels have emerged as a versatile platform for high‐performance molecular and solute‐solute separation [1, 2, 3]. Over the past decade, a broad family of two‐dimensional (2D) nanosheets‐including graphene oxide (GO), MXenes, transition metal dichalcogenides (TMDs), and various inorganic layered solids have been successfully assembled into membranes featuring sub‐nanometer channel networks [4, 5, 6]. These laminar membranes provide unique transport pathways and enable selective sieving arising from their ordered interlayer structures. Among these materials, clay‐based nanosheets, represented by vermiculite (Vm), are gaining increasing attention because of their natural abundance, low cost, scalable exfoliation, chemical robustness, and intrinsically confined interlayers [7, 8, 9]. Clay nanosheet membranes have demonstrated great potential in ion separation [10, 11], solvent purification [12, 13], and osmotic energy conversion [14]. However, their intrinsic interlayer spaces, although structurally well‐defined, are often limited by modest selectivity or insufficient transport rates [15], due to the non‐porous properties on the nanosheet surface, relative torturous transport pathways and the relatively weak electrostatic potential in natural clay layers and spatially distribution, especially when applied to challenging separations such as Li^+^/Mg^2+^, where the hydrated radii are similar but hydration energies differ significantly.

In laminar membranes, ion transport is governed by a combination of size exclusion, Donnan exclusion, and specific interactions between hydrated ions and channel surfaces [16]. Within this framework, precise engineering of interlayer spacing and fine‐tuning of the interlayer chemical microenvironment are essential to optimizing both the permeability and selectivity. Numerous strategies have been explored to tailor these features, including interlayer crosslinking [10], organic or ionic intercalation [17, 18], structural confinement [19], and surface functionalization [20]. Despite these progresses, using functional 2D porous nanomaterials to program interlayer microstructures remains underexplored, even though such materials intrinsically offer long‐range structural modulation, in‐plane sub‐nanopores, and tunable electrostatic characteristics. In particular, 2D MOF nanosheets stand out because their crystalline pore networks, metal‐cluster coordination environments, and precisely adjustable charge distributions uniquely position them for selective ion transport, a capability difficult to achieve with amorphous or organic intercalants.

Recently, 2D metal–organic framework (MOF) nanosheets have emerged as ideal building blocks for laminar channel regulation [21, 22]. However, directly assembling MOF nanosheets into free‐standing membranes remains challenging due to their limited lateral size, poor film‐forming ability, and structural fragility, which often result in discontinuous domains or mechanical instability under operational conditions. When integrated with a stable 2D laminate, MOF nanosheets can function either as interlayer spacers, tailoring through‐plane transport pathways, or as channel surface‐confined modifiers, locally adjusting the chemistry and electrostatic environment of channel openings, compared with small‐molecule intercalants [23, 24]. But how these two confinement modes, interlayer and surface‐level regulation, govern selective cation transport remains insufficiently understood. In particular, the mechanism difference between changing the internal interlayer spacing and modifying the surface‐accessible nanochannels has not been systematically explored. More importantly, MOF nanosheets can provide a degree of pore‐size precision, coordination specificity, and charge controllability that directly matches the energy barrier difference and steric requirements of Li^+^/Mg^2+^ separation.

**FIGURE 1:**
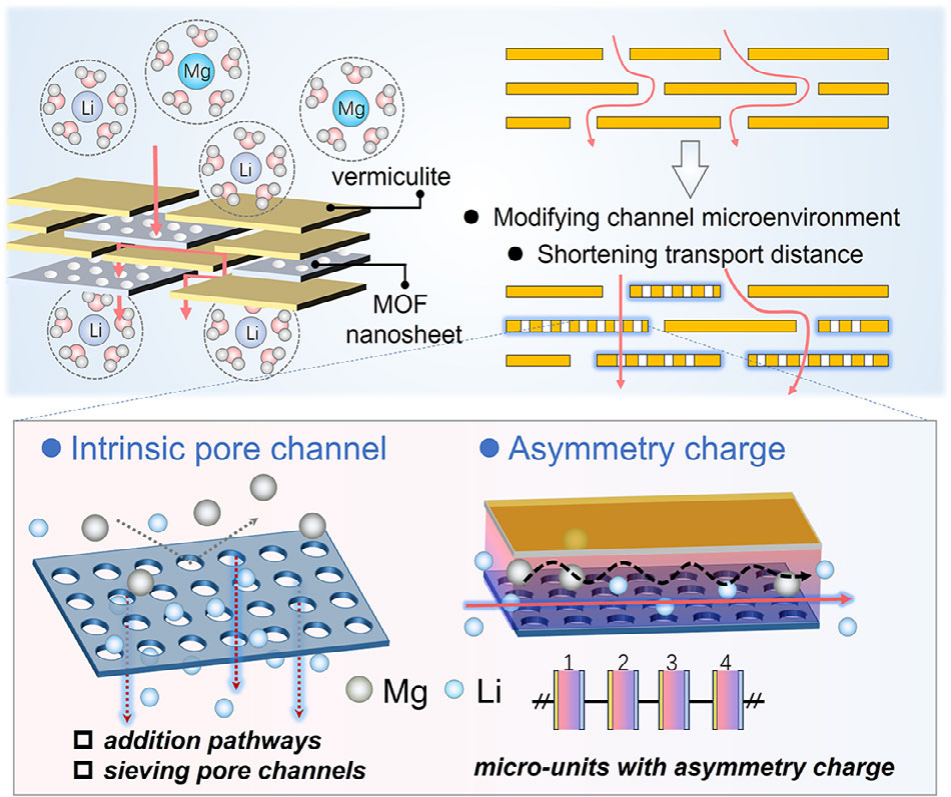
Schematic diagram of an assembly heterogeneous membrane between vermiculite and MOF nanosheets and the possible mass transport mechanism through the heterogeneous laminar structure.

Herein, to achieve high‐efficient Li^+^/Mg^2+^ separation, we employ vermiculite nanosheets as a low‐cost laminar scaffold and integrate 2D Zr‐MOF nanosheets with abundant surface pores constructed from the hexazirconium clusters and BTB linkers, ZrBTB (BTB = 1,3,5‐tri(4‐carboxyphenyl) benzene) via controlled confinement strategies (Figure 1). The introduction of ZrBTB nanosheets with rigid, planar structure can reorganize the interlayer structure with improved channel continuity and high‐efficiency. In addition, the intrinsic charge environment of ZrBTB nanosheets strengthens the charge density and reconstructs the charge distribution inside the interlayer channels, intensifying the exclusion of divalent ions. The optimum membranes achieve a unique combination of increased Li^+^ permeability and significantly enhanced Li^+^/Mg^2+^ selectivity, overcoming the typical permeability‐selectivity trade‐off observed in laminar membranes. To deepen mechanistic understanding, we further employ CuTCPP MOF (TCPP = tetrakis (4‐carboxyphenyl) porphyrin) with the flexible porphyrin macrocycle and different charge distribution compared to ZrBTB nanosheets, enabling to investigate the effects of 2D MOF types on the ion transport pathways. The comparison reveals that ZrBTB drives a selectivity‐dominant interlayer confinement, whereas CuTCPP induces a flux‐dominant channel space manipulation, establishing a clear structure‐performance relationship for MOF‐clay hybrid membranes.

## 2 Results and Discussion

**FIGURE 2:**
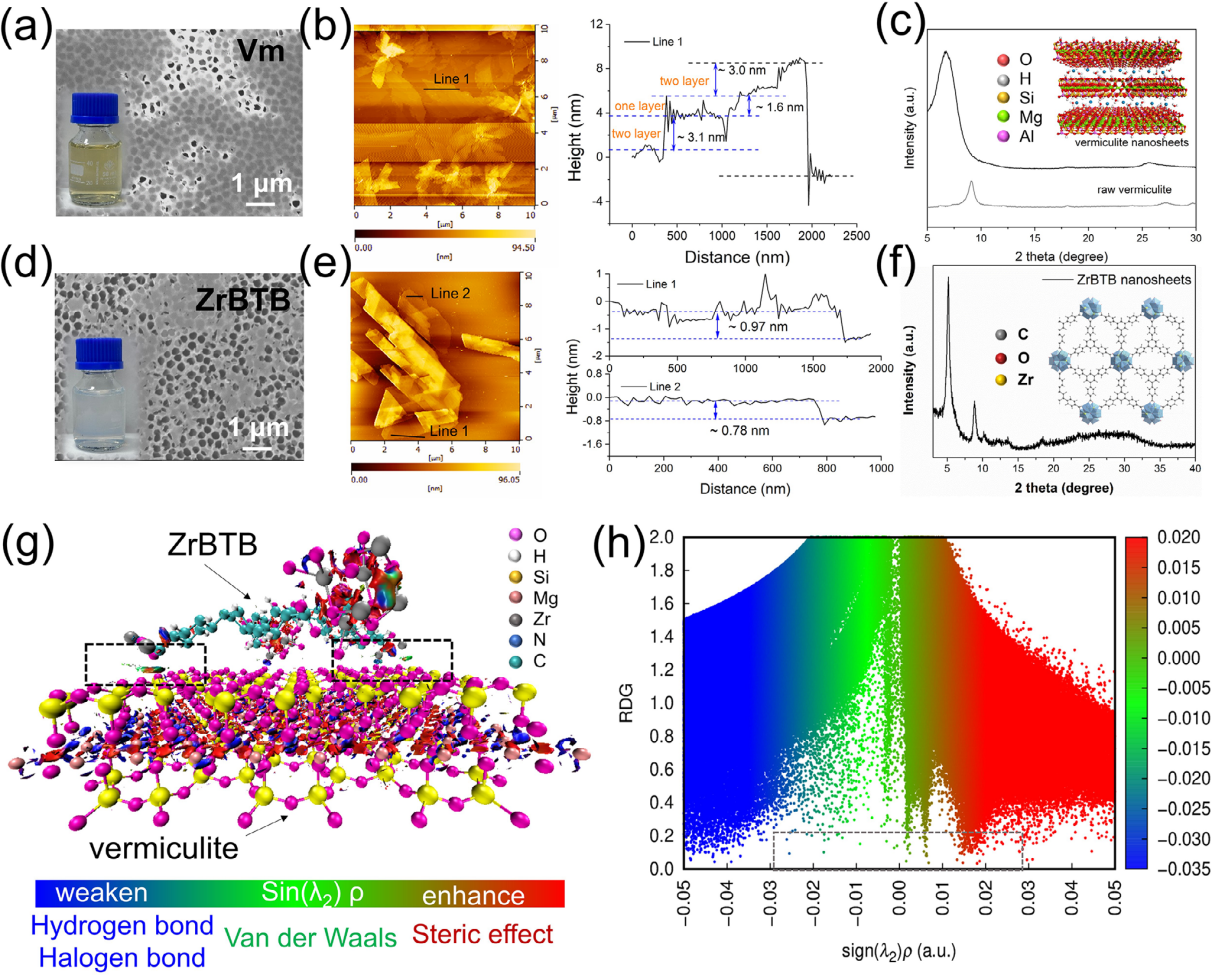
(a) SEM image (Inset is a digital photo of Vm aqueous solution) and (b) AFM image of Vm and corresponding height profile. (c) XRD patterns of exfoliated Vm nanosheets (Inset is an illustration of vermiculite). (d) SEM image (Inset is a digital photo of ZrBTB aqueous solution) and (e) AFM image of ZrBTB nanosheet and corresponding height profile. (f) XRD patterns of exfoliated ZrBTB nanosheets (Inset is an illustration of the ZrBTB MOF structure). (g) 3D isosurface and (h) 2D RDG plots illustrating noncovalent interactions at the ZrBTB‐vermiculite interface. Blue, green, and red regions represent attractive hydrogen bonding/electrostatic interactions, van der Waals interactions, and steric repulsion, respectively.

Vermiculite, a typical member of the 2:1 layered silicate family, is structurally composed of corner‐sharing Si‐O tetrahedra and Al‐O octahedra (Figure 2a). To obtain high‐quality 2D building units, bulk Vm was exfoliated through a two‐step ion‐exchange process (Figures S1 and S2), which effectively weakened the interlayer electrostatic interactions. As shown in Figure 2a–c, the ultrathin vermiculite nanosheets with lateral dimensions of several micrometers were successfully exfoliated and possessed a thickness of ∼1.6 nm, corresponding to a few‐layer structrue. These nanosheets are well dispersed in water, providing ideal precursors for fabricating laminate membranes. In pristine Vm membranes, ion transport dominantly occurs through (i) the edge gaps between adjacent nanosheets and (ii) the interlayer space formed by face‐to‐face stacking (Figure 1). Both pathways are highly tortuous, leading to intrinsically inefficient mass transport. To engineer more efficient transport nanochannels, considering the porous structure on the surface of MOF nanosheets, we synthesized a type of Zr‐MOF nanosheets consisting of the coordination between hexazirconium clusters and BTB linkers through the solvent‐thermal method (Figure 2d) [25]. The obtained ZrBTB nanosheets also possessed a lateral size comparable to Vm and an ultrathin thickness of ∼0.78–0.97 nm (Figure 2d,e; Figure S3), allowing them to be well integrated into Vm layers without introducing macroscopic defects. Further X‐ray diffraction (XRD), X‐ray photoelectron spectroscopy (XPS), and fourier‐transform infrared spectroscopy (FTIR) also confirmed the successful formation of crystalline ZrBTB nanosheets and the preservation of their coordination environment after exfoliation (Figure 2f; Figures S4 and S5). In addition, ZrBTB nanosheets exhibit excellent aqueous stability, and their concentration can be precisely tuned (Figure S6).

**FIGURE 3:**
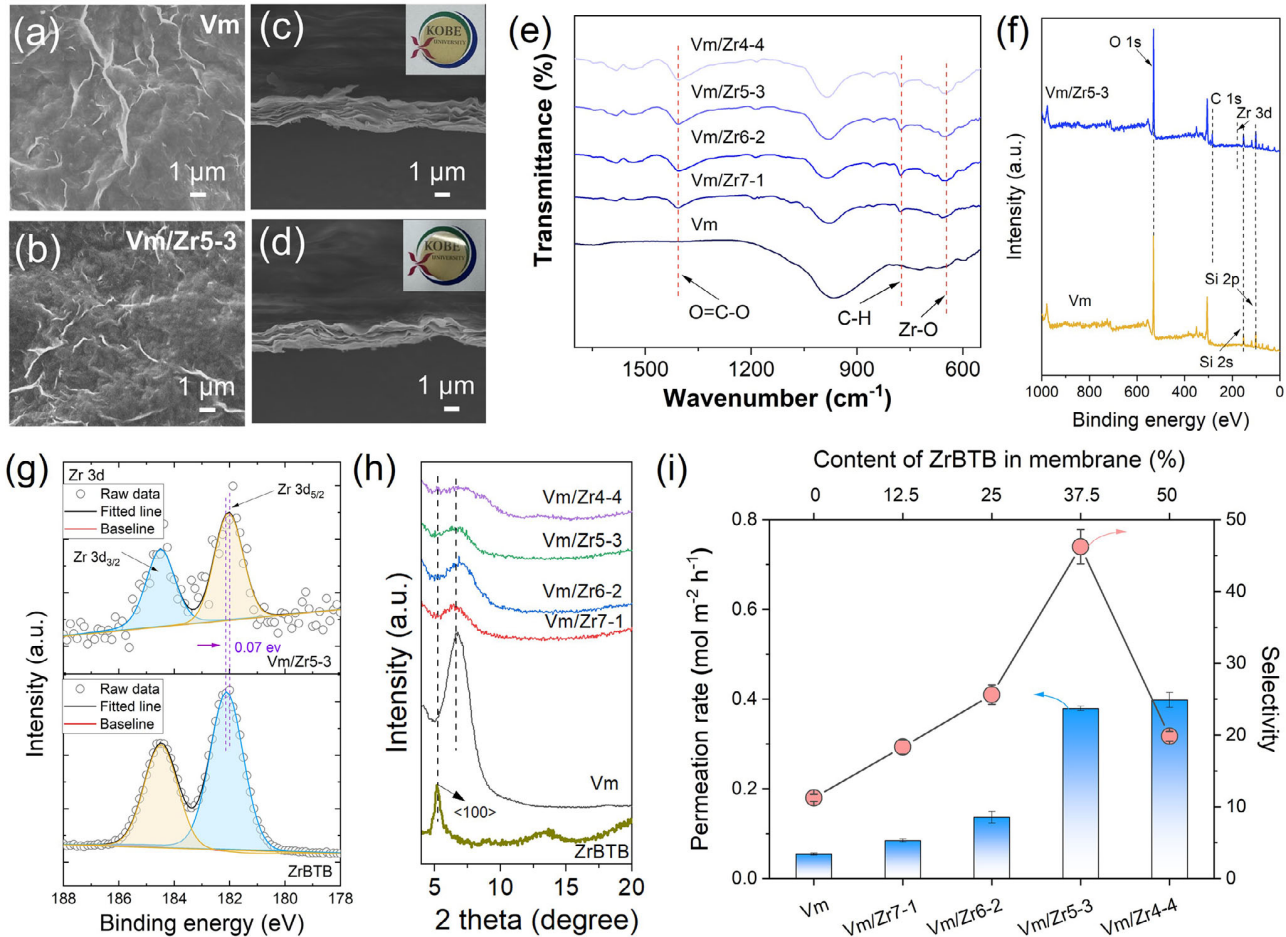
SEM image of surface morphologies of (a) Vm and (b) Vm/Zr5‐3 and cross‐section images of (c) Vm and (d) Vm/Zr5‐3. (e) FTIR spectra of Vm and Vm/Zr composite membranes with different ratio between Vm and ZrBTB nanosheets. (f) Full‐scan XPS spectra of Vm and Vm/Zr5‐3. (g) Zr 3d fitting curves of Vm/Zr5‐3 and ZrBTB powder. (h) XRD patterns of Vm, Vm/Zr composite membranes and ZrBTB membrane. (i) Ion permeation rate and selectivity of Vm/Zr membrane with different assembly ratio.

Furthermore, the interfacial interaction between ZrBTB and vermiculite nanosheets was investigated using NCI‐RDG analysis, as shown in Figure 2g,h. The results indicated that ZrBTB nanosheets are locally anchored onto the vermiculite surface through spatially confined interfacial interactions, including the coexistence of attractive interactions (mainly hydrogen bonding and van der Waals forces), together with steric repulsion at the Vm‐ZrBTB interface. The attractive interactions stabilized the heterogeneous interlayer assembly, while the steric repulsion from the rigid ZrBTB framework suppressed excessive interlayer collapse and preserved the continuity of the lamellar nanochannels. In addition, the UV–vis spectra of Vm/ZrBTBF composite nanosheets showed that the characteristic absorption bands of ZrBTB were well preserved after assembly with Vm, confirming the structural integrity of the MOF nanosheets (Figure S7). Notably, the absorption intensity and band shape exhibit a strong dependence on the Vm/ZrBTB ratio and deviate from a simple linear superposition of the individual components. This behavior indicated significant interfacial interactions between Vm and ZrBTB nanosheets rather than physical mixing. Combined with the simulation results, these observations demonstrate that the Vm‐ZrBTB interaction is sufficient to stabilize heterogeneous interlayer structures and spatially confined enough to maintain well‐defined ion transport pathways. Meanwhile, particle size measurements showed that the average hydrodynamic size of the mixed Vm/ZrBTB dispersion increased compared to the individual nanosheet dispersions (Figure S8), indicating the formation of a heterogeneous composite structure through interfacial interaction. Notably, despite the increased particle size, the mixed dispersion remained highly uniform and still exhibited the Tyndall effect, demonstrating good colloidal stability without macroscopic aggregation. Such a combination of interfacial coupling and dispersion stability facilitates the cooperative assembly of Vm and ZrBTB nanosheets into well‐defined heterogeneous laminar membranes. Such size compatibility and aqueous stability facilitate their co‐assembly. Through the vacuum‐filtration process, the pristine Vm and Vm/ZrBTB (Vm/Zr) membranes were fabricated, respectively, with the amount of nanosheets used remaining consistent (Figure S9). According to the SEM images shown in Figure 3a,b and Figure S10, the incorporation of ZrBTB nanosheets does not change the characteristic surface wrinkles or the overall membrane appearance, and the whole membrane still had similar color to the pristine Vm membrane. With identical deposition amounts, the membrane thickness also remains nearly unchanged (Figure 3c,d; Figure S10), retaining a typical lamellar 2D architecture. FTIR and XPS results confirmed the successful integration of ZrBTB within the Vm framework, evidenced by the appearance of Zr‐O vibrations and detectable Zr signals (Figure 3e,f). Meanwhile, the energy‐dispersive X‐ray spectroscopy (EDX) analysis also provided more element information to support the successful assembly and the uniform distribution of ZrBTB nanosheets on the membrane interlayer and surface (Figures S11–S13). Surface distribution of ZrBTB nanosheets on the membrane also slightly increased the membrane roughness (Figure S14). Further analysis of high‐resolution Zr 3d spectra showed the valence state of Zr increased 0.07 eV, which can be attributed to the formation of new coordination around Zr (Figure 3g). Due to the existence of an exposed O atom on the surface of Vm nanosheets, during the assembling process, the Zr will form a new coordination structure with the O atom of Vm [26, 27]. These interfacial coordination bonds enhance the structural stability of the assembled laminate.

It is noted that assembly with ZrBTB nanosheets had no significant influence on the whole membrane interlayer spacing. According to the XRD results shown in Figure 3h, the main characteristic peak attributed to the Vm had a negligible shift. But further increasing the content of ZrBTB in Vm/Zr membrane can make it possible to detect the signal of ZrBTB attributed to the <100> plane by comparing with the XRD pattern of pristine ZrBTB membrane. Notably, this peak in Vm/Zr4‐4 appeared broadened and reduced in intensity, indicating partial disruption of long‐range MOF ordering due to confinement within the Vm lamellae. This observation also confirmed that ZrBTB nanosheets are structurally preserved but spatially constrained by vermiculite, rather than restacked into bulk‐like MOF domains. Interestingly, the characteristic <001> reflection of pristine Vm, located at ∼ 6.7° with d = 1.32 nm, shows no appreciable shift in peak position after introducing low to moderate amounts of ZrBTB nanosheets, indicating that the average interlayer spacing remains essentially unchanged. However, a significant change in peak magnitude and shape is observed: the <001> intensity progressively decreases, accompanied by significant peak broadening with the increase of ZrBTB loadings. This suggested a possible disruption of the long‐range lamellar order. This structural change revealed that ZrBTB incorporation reorganized the Vm lamellar framework primarily by disrupting long‐range order and generating heterogeneously modified nano‐domains, rather than by producing a homogeneous geometrical expansion.

To further validate the effectiveness of the heterogeneous lamellar architecture for precise ion separation, the ion‐selective permeation performance of the prepared membranes was systematically evaluated. For pristine Vm, there was a balance between membrane thickness and separation performance (Figure S15). Then, as shown in Figure 3i, we first evaluated the influence of the Vm/Zr assembly ratio. With the increase of assembly ratio, the ZrBTB content in the whole membrane gradually increased, and both the corresponding Li^+^ permeation rate and the Li^+^/Mg^2+^ selectivity gradually increased, indicating that the introduction of porous ZrBTB nanosheets effectively promoted ion transport and enhanced ion sieving. When the assembly ratio was 5:3, the Vm/Zr5‐3 composited membrane showed the superior Li^+^/Mg^2+^ separation performance, where the Li^+^ permeation rate was 0.379 mol m^−2^ h^−1^ and the Li^+^/Mg^2+^ selectivity reached around 46.2. Further increasing the ZrBTB fraction, however, resulted in a decline in selectivity. This trend is consistent with the structural analysis above: excessive ZrBTB disrupts long‐range lamellar order, weakens interlayer confinement, and undermines the intrinsic sieving capability of the Vm framework, which simultaneously decreases the permeability and selectivity.

To gain deeper mechanistic insight into the role of interlayer heterogeneity, we synthesized another class of 2D MOF nanosheets, CuTCPP (TCPP = TCPP = tetrakis (4‐carboxyphenyl) porphyrin) by the solvent thermal method [28]. Similarly, the CuTCPP nanosheets also possessed the 2D typical characteristics, stable dispersibility, and intrinsic MOF properties (Figures S16–S20). After intercalation into vermiculite, the resulting Vm/Cu membrane maintained the lamellar architecture with slightly increased surface roughness, and multiple characterizations confirmed the successful assembly of CuTCPP within the interlayers (Figures S21–S24). Importantly, XPS analysis revealed clear changes in the Cu 2p3/2 coordination environment upon integration with Vm, indicating the formation of a stable heterogeneous interlayer, similar to the behavior observed for ZrBTB (Figures S25 and S26). Moreover, the XRD patterns showed progressive peak shifting and broadening with increasing CuTCPP loading (Figure S27a), indicating enlarged interlayer spacing and enhanced structural disorder. The assembled Vm/Cu membrane also showed enhanced Li^+^/Mg^2+^ separation performance compared to the pristine Vm membrane (Figure S27b).

**FIGURE 4:**
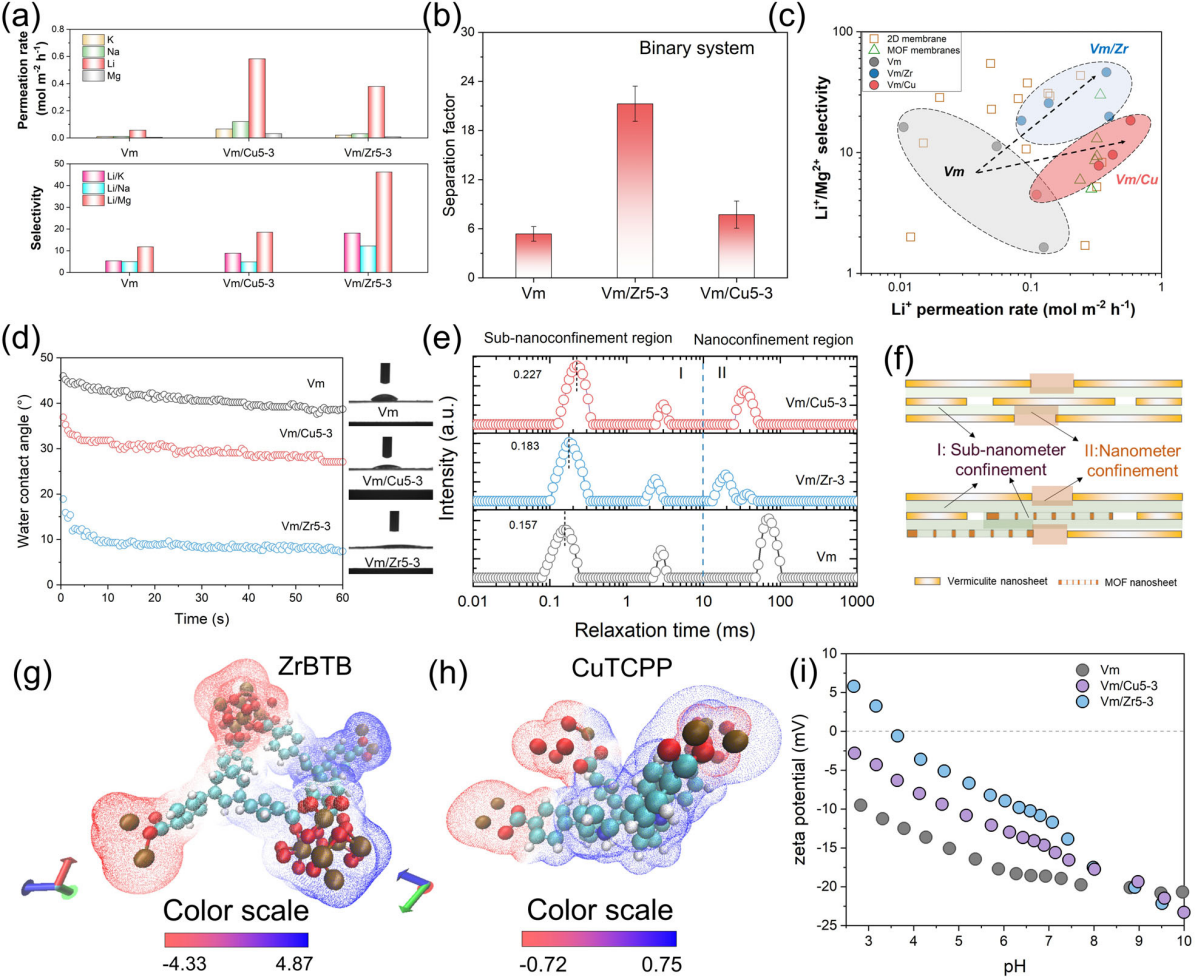
(a) Ion permeation rate and selectivity of Vm, Vm/Zr5‐3, and Vm/Cu5‐3 membranes. (b) The Li^+^/Mg^2+^ selectivity of Vm, Vm/Zr5‐3, and Vm/Cu5‐3 membrane under a binary feed system (0.1 M LiCl + 0.1 M MgCl_2_). (c) Performance comparison of Li^+^ permeation rate and Li^+^/Mg^2+^ selectivity of this work with the reported literatures. (d) Water contact angle curves and (e) LFNMR curves of Vm, Vm/Zr5‐3, and Vm/Cu5‐3 membrane, respectively. (f) Illustration of sub‐nanometer confinement and nanometer confinement region in Vm and Vm/MOF membrane. Electrostatic potential surface of selected (g) ZrBTB and (h) CuTCPP unit. (i) Surface zeta potential of Vm, Vm/Zr5‐3, and Vm/Cu5‐3 membrane, respectively.

Based on the same assembly ratio, we compared the ion separation performance of Vm/Cu and Vm/Zr membrane to gain more understanding about MOF chemistry, as shown in Figure 4a and Figure S28. Incorporating either MOF nanosheet significantly enhanced ion permeation, with the most prominent improvement observed in the Li^+^ flux, confirming that porous MOF domains can provide additional, low‐resistance ion migration pathways. In addition, both MOFs contributed to Li^+^/Mg^2+^ selectivity through their intrinsic pore chemistry; however, their functional influence differed. CuTCPP enhanced Li^+^ permeation more substantially (∼10‐fold relative to pristine Vm), whereas ZrBTB brought a more significant improvement in Li^+^/Mg^2+^ selectivity (∼4‐fold), compared to ∼1.6‐fold for CuTCPP. These contrasting behaviors show that 2D MOF intrinsic properties will influence the ion permeability and selectivity. In addition, when the feed system was changed to a binary Li^+^‐Mg^2+^ mixture, the membranes retained high selectivity despite the presence of strong interionic competition (Figure 4b), further verifying the robustness of the heterogeneous lamellar pathways. Compared with previously reported Li^+^/Mg^2+^ separation membranes operating in diffusion‐driven systems, our Vm/MOF heterogeneous membranes exhibit competitive Li^+^/Mg^2+^ separation performance (Figure 4c; Table S1). Importantly, interlayer spacing had negligible change even though at wet condition (Figure S29) for both Vm and Vm/Zr5‐3 membrane, due to the self‐confinement effect from Vm [11]. Briefly, these results confirm that constructing nano‐confined interlayer heterogeneity with 2D MOF nanosheets is an effective strategy for engineering high‐performance Li^+^/Mg^2+^ separation membranes.

To elucidate how heterogeneity‐guided interlayer engineering modulates ion‐selective transport, a series of structural, chemical, and transport characterizations were conducted. First, we found that incorporating ZrBTB nanosheets significantly reduced the membrane contact angle (Figure 4d), which can be attributed to the intrinsic hydrophilicity and strong capillary effect of ZrBTB frameworks. The decreased contact angle indicates enhanced surface wettability and more efficient water infiltration into the interlayer nanochannels, which is essential for activating hydrated‐ion transport in laminar membranes. Well‐hydrated channels effectively reduce the interfacial energy barrier for ion entry and suppress local dehydration penalties, thereby facilitating continuous and stable ion migration. Nitrogen adsorption‐desorption measurements reveal that pristine ZrBTB nanosheets possess a dominant pore size centered at approximately 0.6 nm, whereas CuTCPP exhibits a significantly larger pore size of about 1.2 nm (Figures S30 and S31), the enhanced capillarity in Vm/Zr membranes is consistent with the formation of narrower and more hydrophilic sub‐nanochannels [26, 29, 30]. Such confined channels preferentially accommodate Li^+^ with lower dehydration energy, while imposing stronger steric and energy penalties on Mg^2+^, whose hydration shell is more rigid. Therefore, the reduced contact angle primarily reflects improved channel hydration and transport accessibility. In addition, the pore size of ZrBTB can contribute to the enhancement of Li^+^/Mg^2+^ selectivity through the size sieving effect. To further verify the reorganization of interlayer pathways, low‐field nuclear magnetic resonance (LF‐NMR) was employed to probe channel‐domain distributions. As shown in Figure 4e,f, the relaxation time (T_2_) distributions can be divided into two different regions: Region I, corresponding to sub‐nanoconfined water strongly restricted within narrow interlayer spaces and sub‐nanometer pores, and Region II, representing nanoconfined water associated with relatively enlarged or newly reorganization channel domains. Upon incorporation of MOF nanosheets, the relaxation populations exhibit a clear redistribution between these two regions, indicating a heterogeneous reconstruction of the interlayer pathways.

Quantitative analysis of the relative peak areas and characteristic relaxation times (summarized in Figure S32 and Table S2) reveals that MOF incorporation systematically changes both the fraction and confinement degree of different channel domains. After introducing the 2D MOF, the relative area increased in Region I compared to pristine Vm, indicating the introduction of a larger proportion of sub‐nanometer sieving channels. Notably, the Vm/Cu membrane shows a larger relative area of Region II, together with a longer relaxation time compared to Vm/Zr, suggesting the reservation of a larger proportion of nanoconfinement channels that facilitate ion transport but are not fully size‐exclusive. This structural change accounts for the significantly enhanced Li^+^ permeability, but only moderate improvement in Li^+^/Mg^2+^ selectivity. In contrast, the Vm/Zr membrane exhibits a significant enrichment of the Region I population. The dominance of sub‐nanoconfined channels with shorter relaxation times indicates the formation of more rigid and size‐selective transport pathways, which are highly favorable for sieving Li^+^ over Mg^2+^. This distinct redistribution of channel domains well explains the different enhancement trends in permeability and selectivity observed for Vm/Cu and Vm/Zr membranes with an intrinsic difference in framework porosity.

Beyond channels structure reorganization, the interlayer charge was also modified. Unlike negatively charged Vm nanosheets, both ZrBTB and CuTCPP nanosheets exhibit positive surface potential (Figure S33). Theoretical simulations of the electrostatic potential (ESP) distributions (Figure 4g,h) further reveal that ZrBTB possesses a significantly broader potential gradient with more intensely localized positive domains compared to the relatively homogeneous and lower‐intensity ESP profile of CuTCPP. After integration, the membrane zeta potential at pH 6.8 shifted from −18.3 mV (Vm) to −14.3 mV (Vm/Cu) and −10.7 mV (Vm/Zr) (Figure 4i). This indicates that while ZrBTB provides stronger localized electrostatic repulsion, CuTCPP achieves a more significant overall surface charge modification. This partial charge change formed the asymmetric charged channel walls, which will show stronger electrostatic exclusion toward divalent Mg^2+^, thereby increasing Li^+^/Mg^2+^ selectivity. Notably, the electrostatic reorganization in Vm/Cu is partially weakened by its larger pore size, which decreases the electrostatic exclusion effect. Furthermore, we tested the lithium‐ion transport properties through Vm and Vm/MOF heterogeneous membrane in LiCl solutions at concentrations ranging from 10^−6^ M to 1 M, where the results also confirmed that the introduction of MOF can modify the charge environment of the Vm interlayer (Figure S34) [11, 31, 32].

**FIGURE 5:**
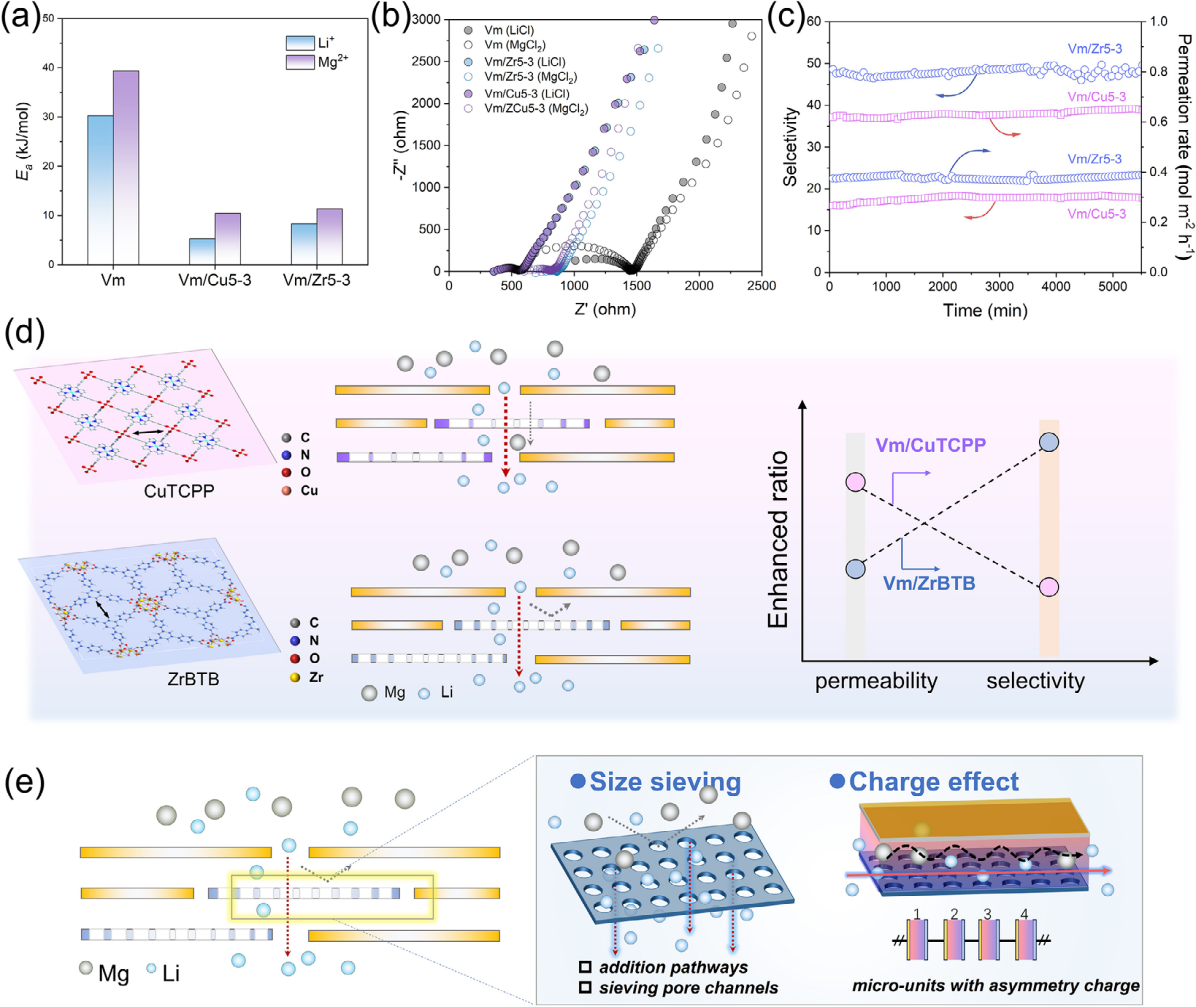
(a) Ion transport energy barrier of Vm, Vm/Cu5‐3, and Vm/Zr5‐3 membrane and (b) Nyquist plots of Vm, Vm/Cu5‐3, and Vm/Zr5‐3 membrane under 0.1 M LiCl and 0.05 M MgCl_2_ solution. (c) Long‐term stability test for Vm/Zr5‐3 and Vm/Cu5‐3 membrane. (d) Schematic diagram of ion‐selective transport through the Vm/Zr and Vm/Cu membrane. (e) Schematic diagram of ion transport through the Vm/MOF membrane govern by the synergistic effect of size sieving and charge effect.

We further calculated the transport activation energies using the Arrhenius equation (Figure 5a; Figure S35). The incorporation of MOF nanosheets significantly reduced the energy barrier for Li^+^ transport while maintaining a high barrier for Mg^2+^, particularly in Vm/Zr membranes. To further confirm the reduction of ion transport resistance induced by porous MOF nanosheets, electrochemical impedance spectroscopy (EIS) measurements were conducted (Figure 5b). Compared with pristine vermiculite membranes, both Vm/Zr and Vm/Cu membranes exhibit significantly reduced high‐frequency intercepts and smaller semicircle diameters, indicating a decrease in membrane resistance after MOF incorporation. This resistance reduction arises from the heterogeneous reorganization of interlayer channels, which introduces additional ion‐accessible pathways and shortens effective transport distance. Notably, Vm/Cu membranes show the lowest overall resistance, consistent with their higher ion permeation rates, whereas Vm/Zr membranes maintain relatively higher resistance toward Mg^2+^ transport, showing stronger size‐ and charge‐based exclusion. These results confirm that MOF‐guided interlayer engineering effectively lowers ion transport barriers while enabling distinct permeability‐selectivity regulation through rational MOF selection. In addition, the membranes demonstrated excellent operational stability, maintaining consistent separation efficiency for over 5500 min (Figure 5c).

Overall, the interplay between heterogeneous channel size and charge defines the different separation behaviors of Vm/Zr and Vm/Cu membranes (Figure 5d). The incorporation of ZrBTB nanosheets, featuring smaller intrinsic pore apertures and moderately positive charge domains, preferentially enriches sub‐nanoconfined pathways with enhanced electrostatic exclusion [20, 33, 34]. This structure significantly strengthens the repulsion toward Mg^2+^, while maintaining accessible transport pathways for Li^+^, thereby enabling highly efficient Li^+^/Mg^2+^ separation dominated by size‐charge cooperative sieving. In contrast, CuTCPP nanosheets possess a larger pore size and weakly charged regions. While these pathways remain selective, their reduced steric constraints and weaker electrostatic repulsion further accelerate monovalent ion transport, resulting in significant permeability enhancement with comparatively limited selectivity.

Importantly, these results show a structure/interlayer charge‐programmable strategy in which ion transport behavior is governed not by a single structural parameter but by the balance between pore‐size heterogeneity and charge heterogeneity (Figure 5e). By rationally selecting MOF nanosheets with tailored intrinsic porosity and electrostatic characteristics, the interlayer microenvironment of 2D laminar membranes becomes a tunable design space. This approach enables targeted optimization toward either selectivity‐dominant or flux‐dominant regimes, effectively alleviating the conventional permeability‐selectivity trade‐off without compromising membrane integrity.

## 3 Conclusions

In this work, we demonstrated a heterogeneity‐driven interlayer engineering strategy to modulate ion transport in 2D vermiculite membranes by incorporating ultrathin ZrBTB nanosheets. Unlike conventional approaches that rely on expanding the interlayer spacing, our results show that ZrBTB nanosheets reorganize the lamellar architecture primarily through disruption of long‐range ordering and creation of diverse sub‐nanoconfinement domains, rather than geometrical expansion. This structural reconstruction, together with partial charge change, simultaneously promotes fast Li^+^ transport while enabling strong steric and electrostatic exclusions on Mg^2+^. The resulting Vm/Zr membranes showed simultaneously high Li^+^ permeation and exceptional Li^+^/Mg^2+^ selectivity, outperforming most reported diffusion‐based membranes. By comparing ZrBTB and CuTCPP‐integrated membranes, we further established a mechanistic framework revealing how pore size and charge state can be independently tuned using MOF nanosheets as programmable interlayer modifiers. Beyond lithium extraction, the concept of interlayer heterogeneity offers new opportunities for developing selective membranes for seawater desalination, Li‐ion battery recycling, and resource recovery from complex ionic environments.

## Conflicts of Interest

The authors declare no conflicts of interest.

## Supporting information




**Supporting File**: advs74597‐sup‐0001‐SuppMat.pdf.

## Data Availability

The data that support the findings of this study are available from the corresponding author upon reasonable request.
