# Supplementary material for: Heterogeneity‐Guided Interlayer Tuning in Vermiculite‐MOF Membranes for Efficient Li+/Mg2+ Separation
Source: Adv Sci (Weinh). 2026 Feb 27;13(26):e74597. doi: 10.1002/advs.74597 (PMC13159124; doi:10.1002/advs.74597)
Supplement: Supplementary file 1 — Supporting File: advs74597‐sup‐0001‐SuppMat.pdf. [file ADVS-13-e74597-s001.pdf]

Supporting Information

Heterogeneity-Guided Interlayer Tuning in Vermiculite-MOF Membranes for Efficient Li^{+}/Mg^{2+} Separation

Liheng Dai[1], Kecheng Guan[1,*], Mengyang Hu[1], Erda Deng[1], Pengfei Zhang[1], Zhan Li[1], Yongxuan Shi[1], Xiao Xu[1], Xueru Yan[1], Hideto Matsuyama[1,2,*]

1 Research Center for Membrane and Film Technology, Kobe University, 1-1 Rokkodai, Nada, Kobe, 657-8501 Japan
2 Department of Chemical Science and Engineering, Kobe University, 1-1 Rokkodai, Nada, Kobe, 657-8501 Japan

*: Corresponding author: Email: matuyama@kobe-u.ac.jp;
guan@people.kobe-u.ac.jp

Materials

Vermiculite bulk was purchased from Artec Co., Ltd., Japan. Methanol, Ethanol, N, N-Dimethylformamide, Copper (II) Nitrate Trihydrate, formic acid and Zirconium (IV) Chloride were FUJIFILM Wako Pure Chemical Industries Co. Ltd. (Osaka, Japan). 1,3,5-tri(4-carboxyphenyl) benzene and Tetrakis (4-carboxyphenyl) porphyrin was purchased from Tokyo Chemical Industry (TCI) Co., Ltd., Japan. Anodized aluminum oxide with 0.2 μm pore size, 25- and 47-mm diameter (AAO, Whatman) was used as substrates to prepare freestanding 2D membrane. NaCl, KCl, LiCl, and $MgCl_2$ were purchased from FUJIFILM Wako Pure Chemical Industries Co. Ltd. (Osaka, Japan). Ultrapure water was obtained using a Milli-Q filtration system (Merck Millipore, Japan) for all the experiments. All chemicals were used without further purification.

Experiment

Preparation of vermiculite nanosheets

The exfoliation of vermiculite (Vm) nanosheets was conducted through two-step ion exchange process according to our previous works[1]. Typically, 1 g of raw vermiculite particles was added to 100 mL of saturated NaCl solution and subjected to refluxing for 24 h. After naturally cooling to room temperature, the solid was collected and washed with deionized water five times. The obtained solid was then redispersed in 100 mL of 2 M LiCl solution and refluxed for an additional 24 h. After completing the second ion-exchange step, the product was filtered and thoroughly washed with deionized water five times. The resulting material was subsequently dispersed in deionized water and ultrasonicated for 10 h to exfoliate the particles into few-layer nanosheets. Finally, the 2D vermiculite nanosheets were collected by centrifugation at 10,000 rpm for 30 min. The obtained concentration of Vm nanosheets was calculated at ~ 2.8 mg/mL.

Preparation of ZrBTB nanosheets

Typically, 30 mg of $ZrCl_4$ and 30 mg of 1,3,5-tri(4-carboxyphenyl) benzene (BTB) were dissolved in a mixed solvent consisting of 15 mL N, N-dimethylformamide (DMF), 2 mL formic acid, and 2 mL deionized water[2]. The precursor solution was sonicated for 5 min and then transferred to an oven at 120 °C for 2 h to facilitate the solvothermal reaction. The resulting product was collected and washed five times with fresh DMF by centrifugation at 8000 rpm for 10 min. The solid was subsequently soaked in DMF overnight, followed by washing with deionized water five times. Finally, the purified ZrBTB was dispersed in water for storage. The concentration was calculated according to the as-obtained membrane weight difference before and after filtering 50 mL ZrBTB solution. The concentration of ZrBTB was ~ 28 mg/L.

Preparation of CuTCPP nanosheets

CuTCPP) nanosheets were synthesized following a previously reported method. Typically, 0.15 mmol, 36.5 mg of $Cu(NO_3)_2·3H_2O$ was dispersed in 40 mL of DMF, while 0.05 mmol, 40.0 mg of TCPP was dissolved in another 40 mL of DMF[3]. The two solutions were combined and ultrasonicated for 30 min to obtain a homogeneous precursor mixture. The mixture was first aged at 25 °C for 1 h and subsequently heated at 80 °C for 4 h. After the reaction, the system was allowed to cool naturally to room temperature over 12 h. The resulting gel-like product was collected by centrifugation (8000 rpm, 10 min) and washed with ethanol more than five times until the supernatant became colorless. The obtained purple solution was stored in ethanol solution for following usage. The concentration was calculated according to the as-obtained membrane weight difference before and after filtrating 50 mL CuTCPP solution. The concentration of CuTCPP was ~34 mg/L.

Preparation of vermiculite/MOF composite membrane

The composite membranes were fabricated using a vacuum filtration method, as shown in Figure S9. Specifically, the mixed dispersion containing Vm nanosheets and MOF nanosheets was filtered onto an AAO support, followed by drying at 40 °C for 24 h. The resulting membrane could be easily peeled off from the AAO substrate to obtain a freestanding membrane. To investigate the influence of the assembly ratio between Vm and MOF nanosheets, the mass of Vm nanosheets varied at 1.6, 2.0, 2.4, and 2.8 mg, while the amount of MOF nanosheets was adjusted to 1.6, 1.2, 0.8, and 0.4 mg, respectively. The prepared composite membranes were denoted as Vm/Zrx-y or Vm/Cux-y, where *x* and *y* correspond to the mass of Vm and MOF nanosheets, respectively. For comparison, a pristine Vm membrane was fabricated following the same procedure in the absence of MOF nanosheets.

Characterization

Field-emission scanning electron microscopy (FESEM; JSF-7500F, JEOL Co. Ltd., Tokyo, Japan) was used to observe the morphologies of nanosheets, and the surface or cross-section morphologies of the Vm, Vm/MOF composite membranes. The element analysis of membrane was conducted by energy-dispersive spectrometry (EDS, Phenom, Thermo Fisher Scientific, USA). Chemical structure and composition were analyzed using X-ray photoelectron spectroscopy (XPS; PHI GENESIS, Physical Electronics, Inc. (PHI), USA) and attenuated total reflectance Fourier-transform infrared (ATR-FTIR, PerkinElmer) spectroscopy. The contact angle testing system (Drop Master 300; Kyowa Interface Science Co., Ltd., Tokyo, Japan) was used to evaluate the membrane surface hydrophilicity. X-ray diffractometer (XRD; D2 PHASER 2ndGen, Bruker, Billerica, MA, USA) was conducted to analyze the membrane structure. The UV-vis spectra (V-650, Jasco International Co., Ltd., Tokyo, Japan) of the CuTCPP and ZrBTB suspensions were recorded in the range of 200–800 nm. The zeta potential of the membrane was determined by the electrokinetic

analyzer (Anton Paar SurPASSTM 3, Austria) using 1 mM KCl as the electrolyte solution. Meanwhile, the zeta potential of vermiculite, ZrBTB and CuTCPP suspensions were also measured using the Malvern Particle Size Analysis. Low-field nuclear magnetic resonance (LF-NMR, MesoMR23-060H-I) was employed to study the molecular dynamics of the water as probe molecules within the Vm and Vm/MOF membranes. Membrane surface roughness and the thickness of nanosheet were further characterized by atomic force microscopy (AFM, Bruker Dimension Icon). Meanwhile, the concentrations of ionic species in permeate solutions for binary system test were precisely measured using inductively coupled plasma mass spectrometry (ICP-MS, Shimadzu).

Ion permeation test

To evaluate the ion separation performance, the membranes were tested using a custom-designed H-type diffusion cell composed of a donating chamber and a receiving chamber. Each chamber was filled with 150 mL of solution, with deionized water in the receiving chamber and the corresponding 0.2 M salt solution in the donating chamber. The membrane was fixed between the two chambers and sealed with epoxy resin, and the effective membrane area was determined using ImageJ software. During the diffusion experiments, both chambers were continuously stirred to minimize concentration polarization. The conductivity of the receiving chamber was monitored in real time using a conductivity meter (D-220C-S, HORIBA, Kyoto, Japan).

The ion permeation rate (P_i) was calculated with the following equation;

$$P_i = \frac{\Delta C_p \times V}{A \times t} \tag{1}$$

where ΔC_p is the change value concentration (mol L^{-1}), V is the volume of the aqueous solution (mL), A is the calculated effective area of the test membrane and t is the recored permeation time.

The ion concentration in the permeate side ($C_{i,p}$) can be calculated according to following equation;

$$C_{i,p} = \frac{\lambda_i}{\Lambda_m} \tag{2}$$

where Λ_m is the molar conductivity (S cm^{-1}/mol L^{-1}), a constant characteristic of a given ion, determined by fitting the conductivity data obtained from salt solutions of known concentrations. λ_i represents the measured ionic conductivity (S/cm).

The ideal ion selectivity was calculated according to equation (3);

$$S_i = \frac{P_i}{P_j} \tag{3}$$

where P_i and P_j were the permeation rate of i and j ion.

Ion transport energy barrier

Ion transport energy barrier can be calculated based on the Arrhenius-type equation;

$$P_i = P_0 e^{-\frac{E_{a,i}}{RT}} \tag{4}$$

$$ln\ (P_i) = \ln\ (P_0) - \frac{E_{a,i}}{RT} \tag{5}$$

where P_0 was the pre-exponential facto, $E_{a,i}$ was the activation energy related to the energy barrier, R was a constant (8.314 x 10^{-3} kJ mol^{-1} K^{-1}), and T was the temperature. $E_{a,i}$ could be evaluated from Arrhenius plots (*In (P_i) vs. 1000/T*).

In detail, the ion permeation rate at different temperature (25, 30, 35, 40 ℃) was conducted and recorded.

Computational details

The reduced density gradient (RDG)-based non-covalent interaction (NCI) method was employed to analyze the non-covalent interactions between vermiculite and two-dimensional metal-organic frameworks (2D MOFs, Zr-BTB). This method enables real-space visualization of weak intermolecular interactions. All-atom molecular dynamics (MD) simulations were performed using BIOVIA Materials Studio 2023 software and a universal force field (UFF) to obtain the equilibrium interface configuration [4]. Energy minimization was performed before the MD simulations to eliminate unfavourable contacts [5].

The system was equilibrated under isothermal-isobaric (NPT) conditions at 298.15 K and 1.0 bar, with temperature control using a Nosé thermostat. The equilibrium MD simulations lasted for 2 ns, and representative snapshots were extracted from the equilibrium trajectory for subsequent electronic structure analysis.

Subsequently, using the ORCA 4.2.0 software package, geometric optimization and single-point energy calculations were performed at the B3LYP-D3/6-31G(d) theoretical level, with Grimme's D3 dispersion correction explicitly introduced to accurately characterize long-range interactions [6-10]. Electrostatic potential (ESP) mapping and NCI-RDG analysis were then performed using the Multiwfn program to elucidate the nature and strength of the interfacial interactions, respectively [11, 12]. The structural model and interaction characteristics were visualized using Visual Molecular Dynamics (VMD, version 1.9.4a53) [13].

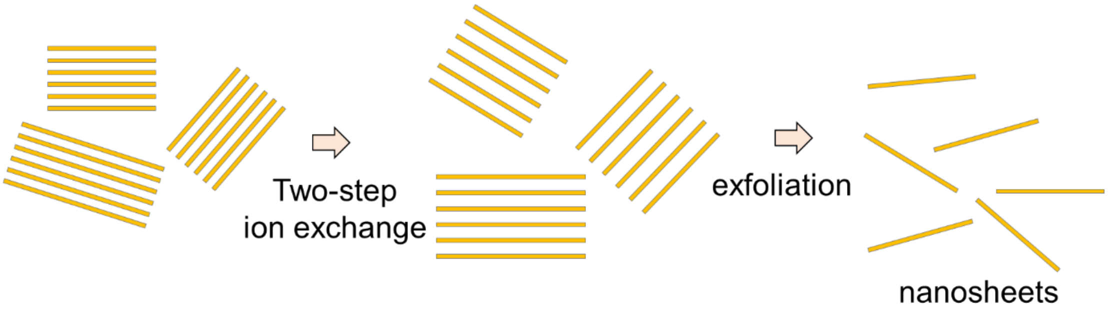

Figure S1. Schematic diagram of exfoliation of vermiculite nanosheets.

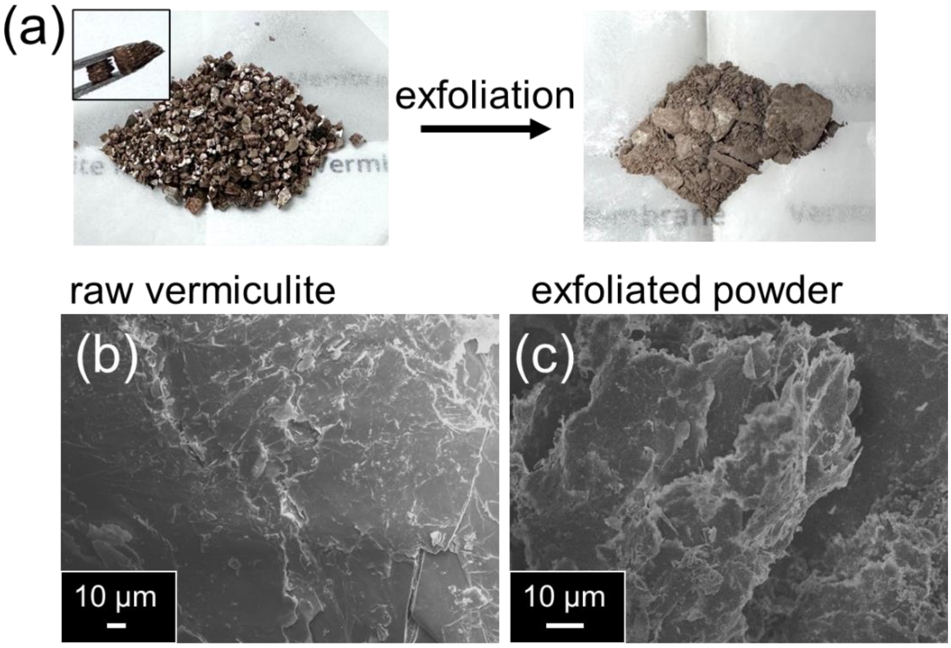

Figure S2. (a) Digital photos of raw vermiculite particles and exfoliated 2D vermiculite powder. SEM images of (b) raw vermiculite and (c) exfoliated vermiculite nanosheets.

According to **Figure S2**, it can be seen that the raw vermiculite possesses the layer structure, and ultrathin nanosheets can be obtained after exfoliation.

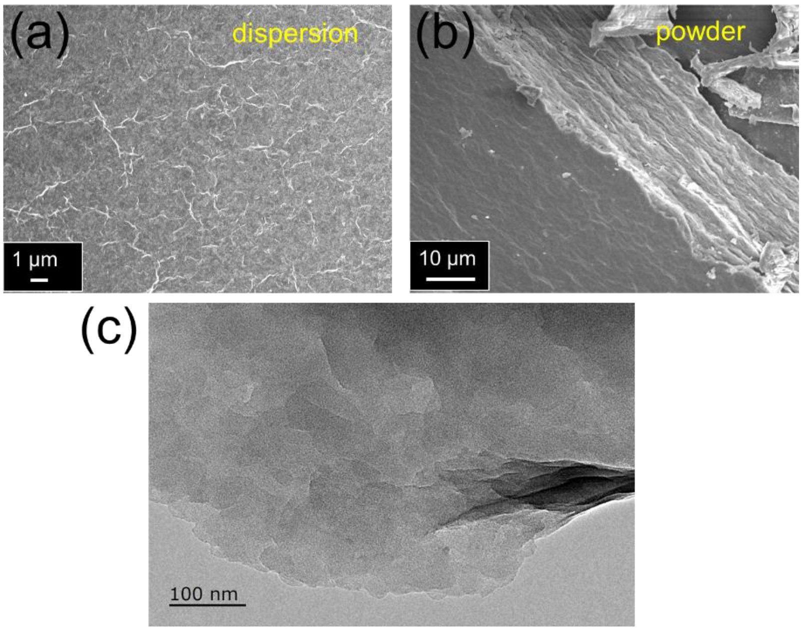

Figure S3. SEM images of (a) ZrBTB nanosheets dispersion and (b) collected ZrBTB nanosheets powder after centrifugation. (c) TEM image of ZrBTB nanosheet.

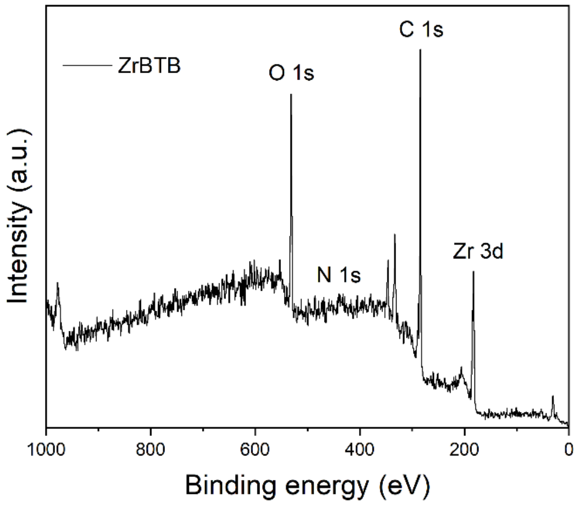

Figure S4. Full-scan XPS spectrum of ZrBTB nanosheets.

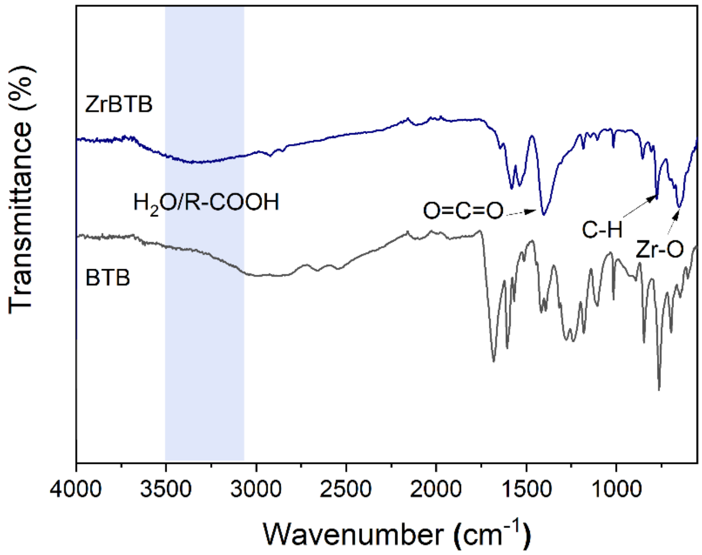

Figure S5. FTIR spectra of ZrBTB nanosheets and BTB monomer.

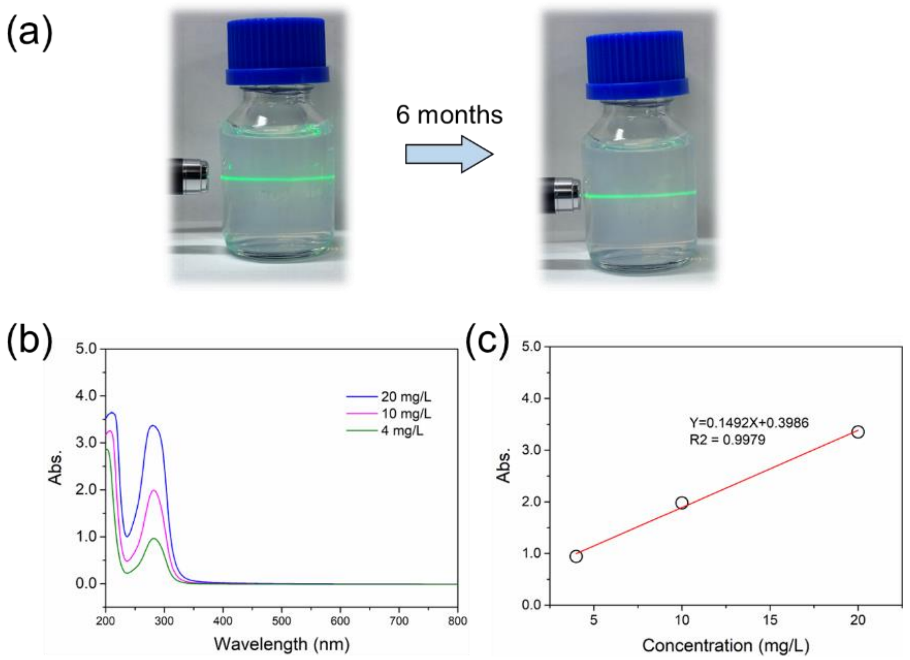

Figure S6. (a) Digital photo of ZrBTB nanosheets solution for six months. (b) UV-vis spectra of ZrBTB solution with different concentration. (c) the relationship between absorbance and concentration of ZrBTB nanosheets solution.

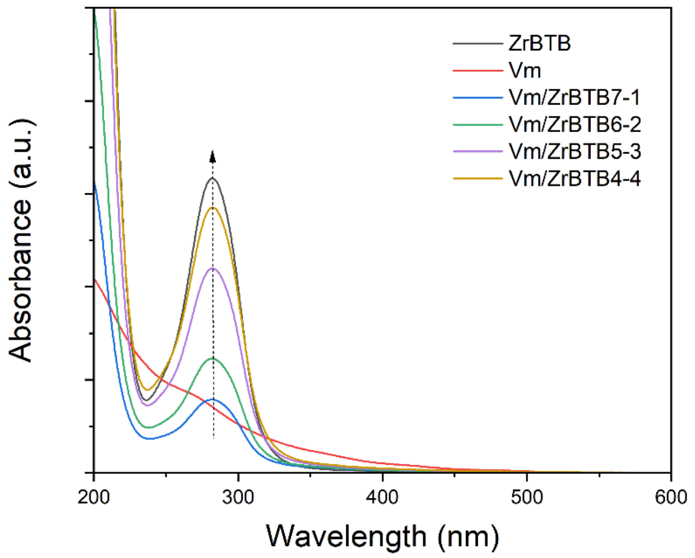

Figure S7. UV-vis curves of ZrBTB nanosheets, Vm nanosheets and Vm/ZrBTB with different assembly ratio. Note: the sample was diluted by 60-folds before test.

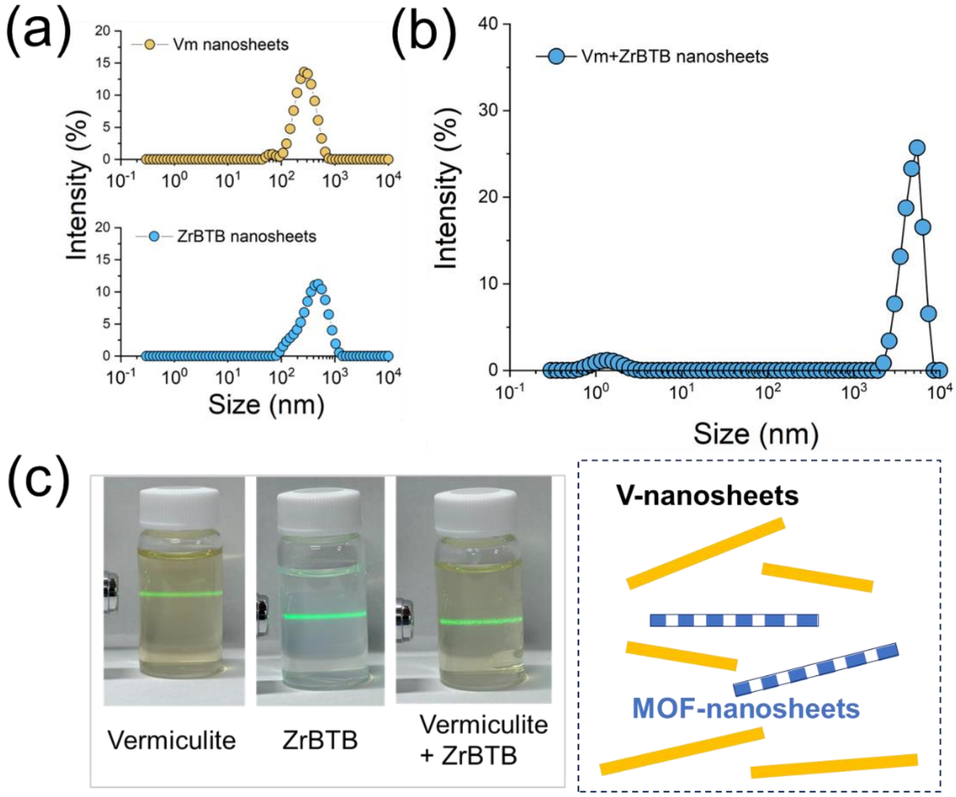

Figure S8. (a) Particle size plots of Vm and ZrBTB nanosheets. (b) Particle size plot of Vm+ZrBTB mixture solution. (c) Digital photos of Vm, ZrBTB and Vm+ZrBTB mixture under laser irradiation and schematic diagram of assembly behavior between Vm and ZrBTB nanosheets.

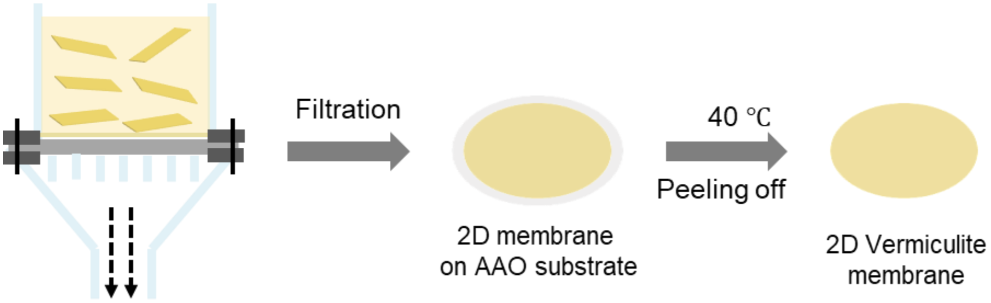

Figure S9. Schematic diagram of membrane fabrication process.

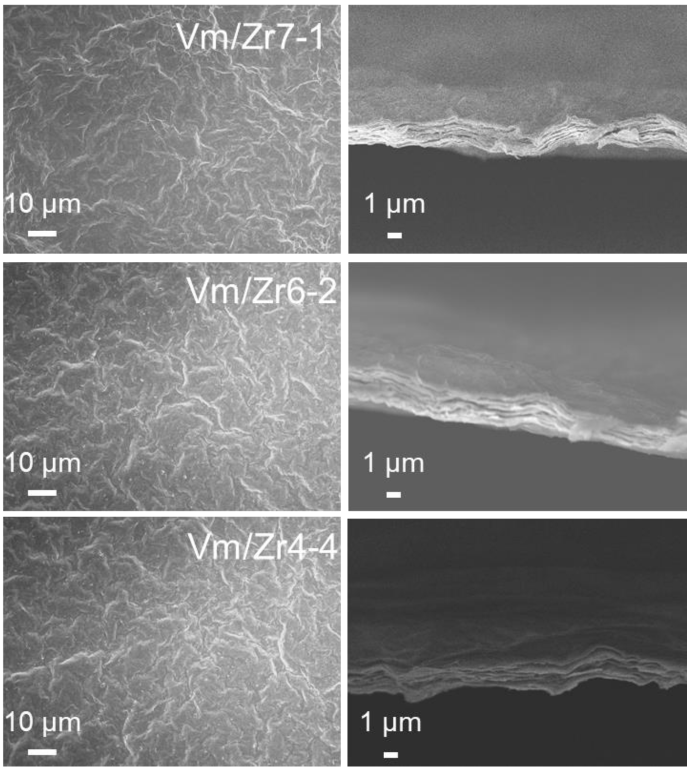

Figure S10. SEM images of surface morphologies and cross-section of Vm/Zr with various assembly ratio.

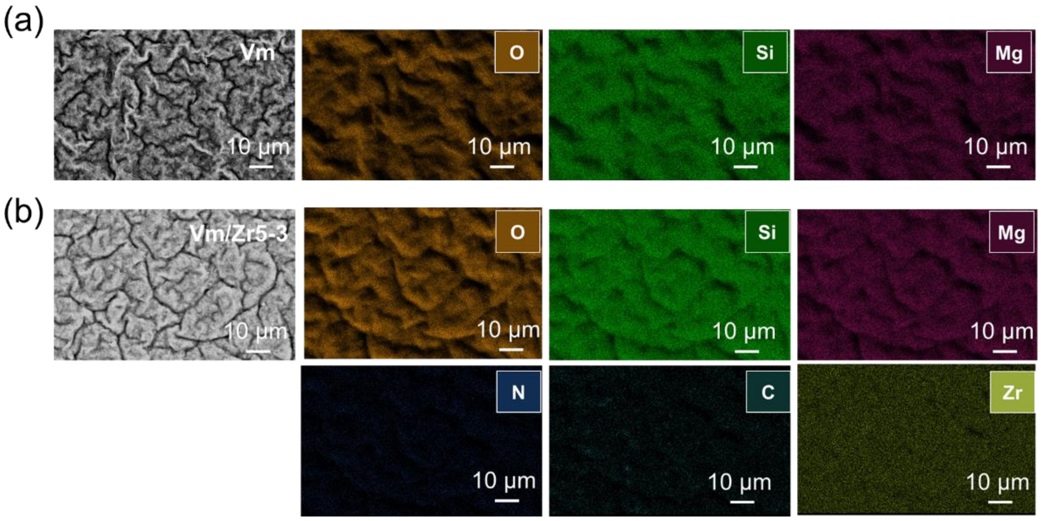

Figure S11. EDX mapping of membrane surface of (a) Vm and (b) Vm/Zr membrane.

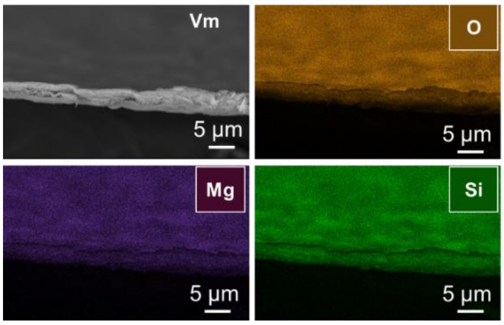

Figure S12. EDX mapping of membrane cross-section of Vm membrane.

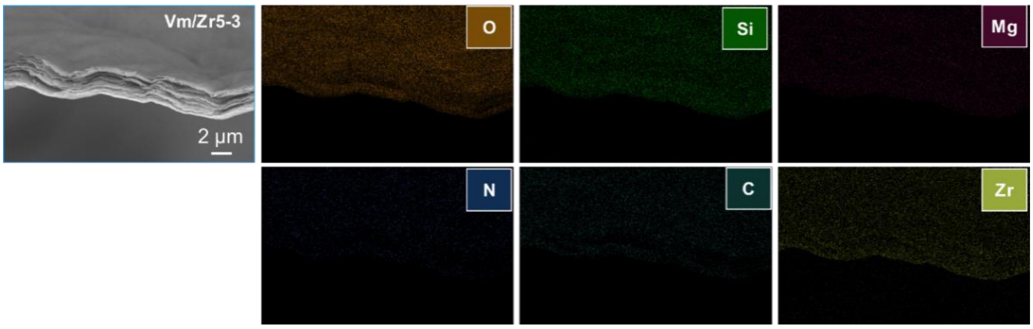

Figure S13. EDX mapping of membrane cross-section of Vm/Zr membrane.

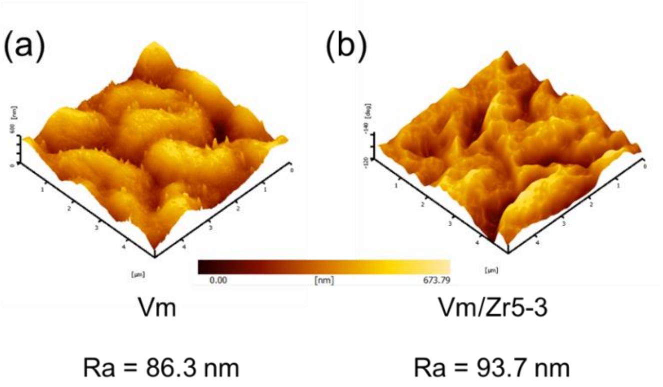

Figure S14. AFM images of (a) Vm and (b) Vm/Zr membrane.

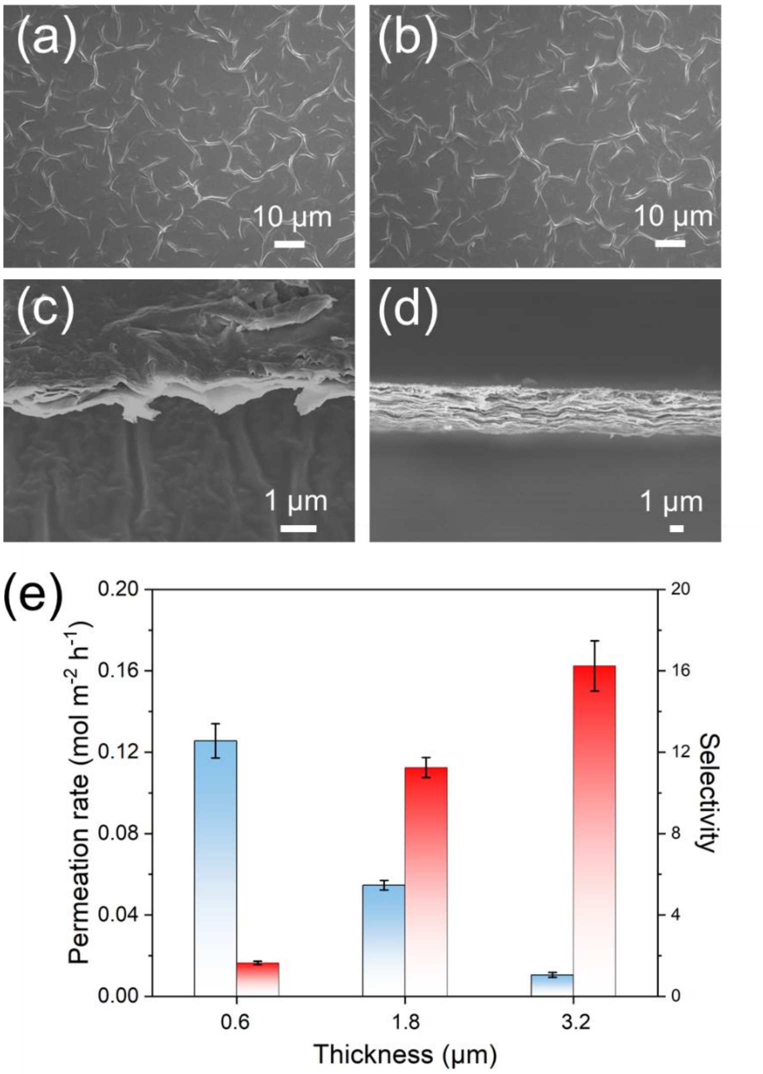

Figure S15. SEM images of (a-b) surface morphologies and (c-d) cross-section of pristine Vm membrane with different membrane thickness. (e) Ion permeation performance of pristine Vm membrane varying different membrane thickness.

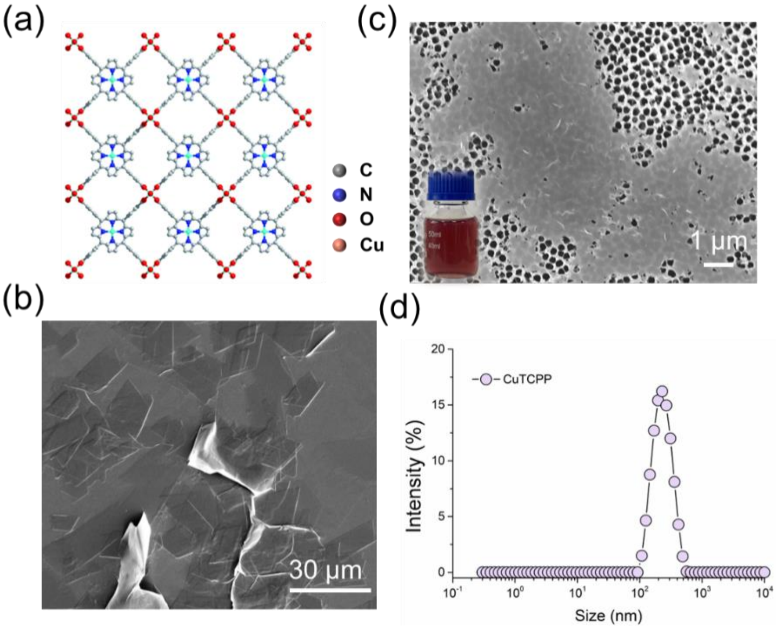

Figure S16. (a) Schematic diagram of CuTCPP MOF structure. (b-c) SEM image and (d) Particle size distribution of CuTCPP nanosheets.

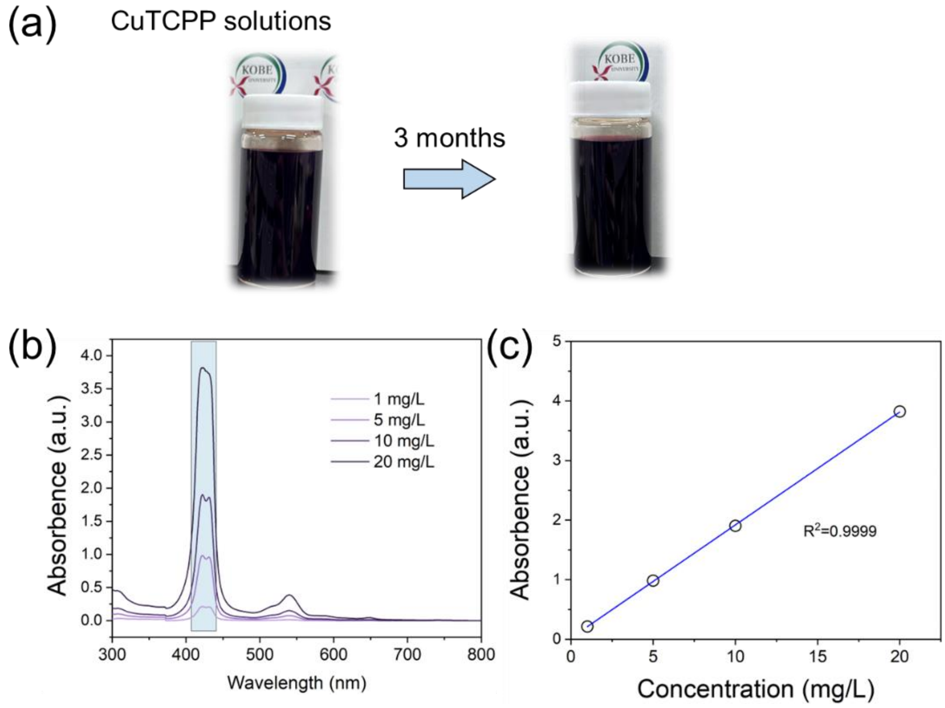

Figure S17. (a) Digital photo of CuTCPP nanosheets solution for three months. (b) UV-vis spectra of CuTCPP solution with different concentration. (c) the relationship between absorbance and concentration of CuTCPP nanosheets solution

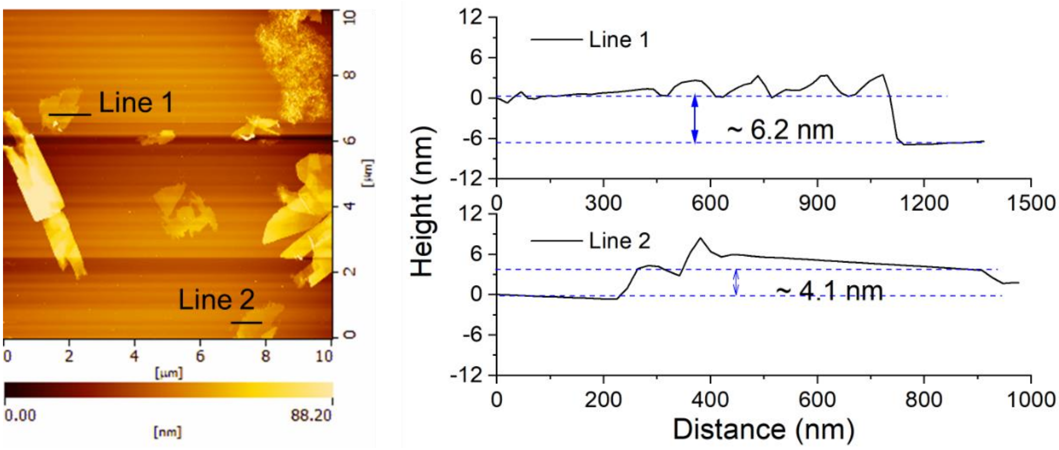

Figure S18. AFM images of CuTCPP nanosheets.

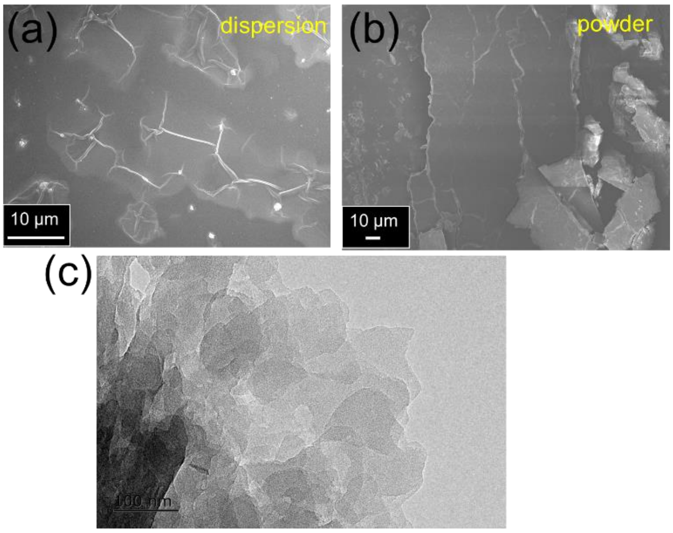

Figure S19. SEM images of (a) CuTCPP nanosheets dispersion and (b) collected CuTCPP nanosheets powder after centrifugation. (c) TEM image of CuTCPP nanosheet.

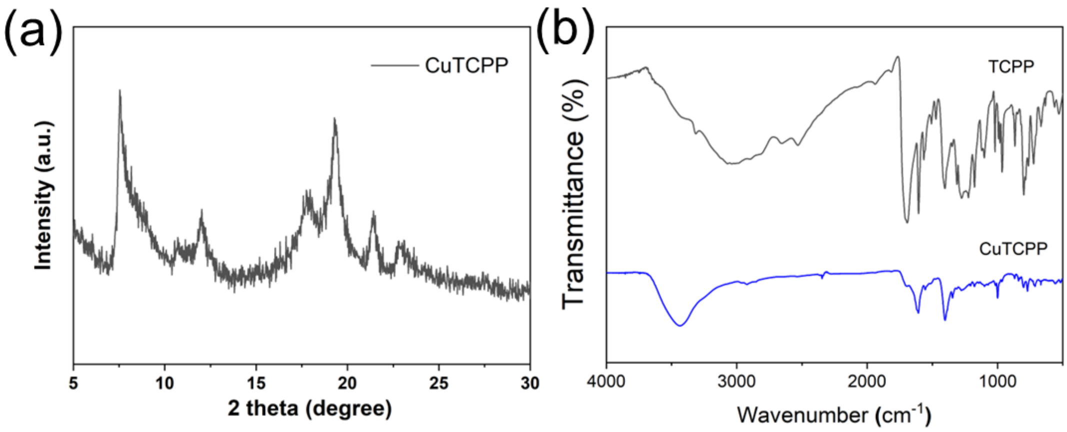

Figure S20. (a) XRD pattern and (b) FTIR spectra of CuTCPP nanosheets.

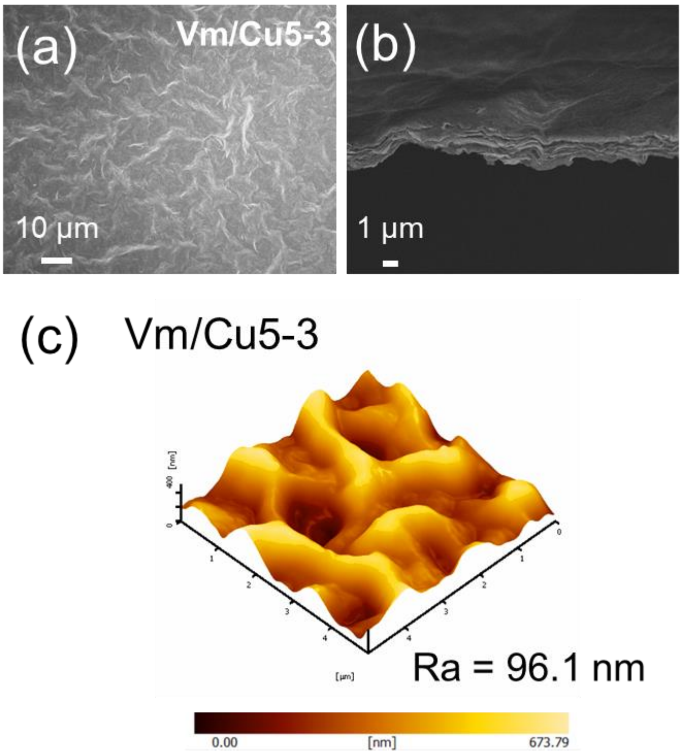

Figure S21. SEM images of (a) surface morphologies and (b) cross-section, and (c) AFM image and surface roughness of Vm/Cu5-3 membrane.

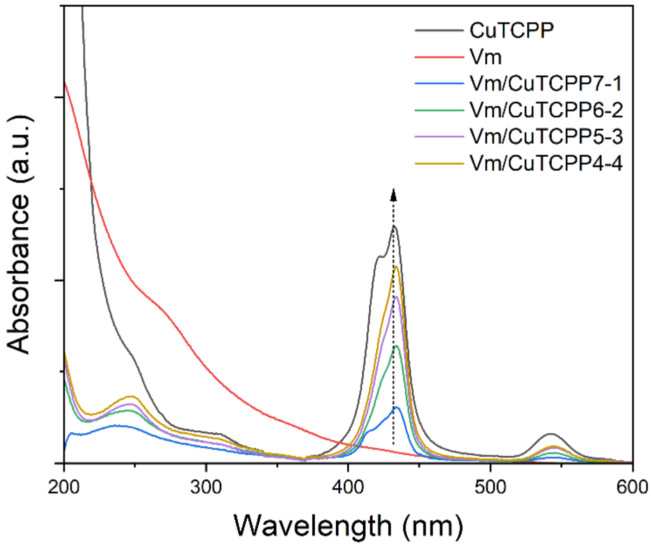

Figure S22. UV-vis curves of CuTCPP nanosheets, Vm nanosheets and Vm/ CuTCPP with different assembly ratio. Note: the sample was diluted by 60-folds before test.

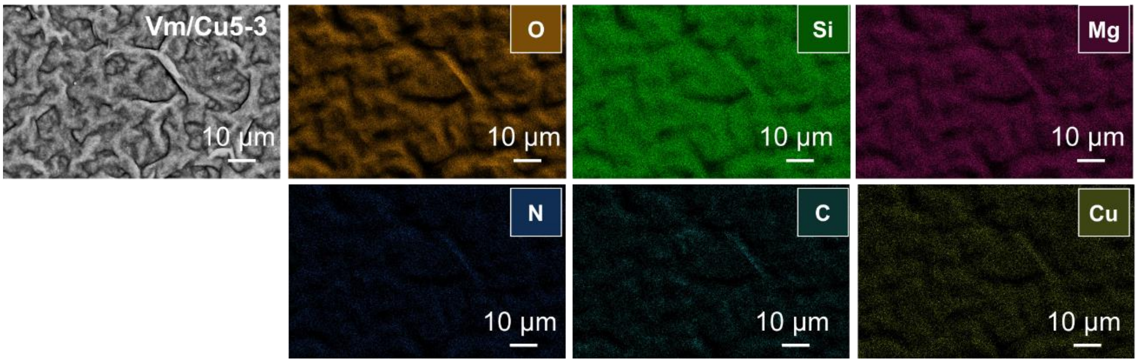

Figure S23. EDX mapping images of surface of Vm/Cu5-3 membrane.

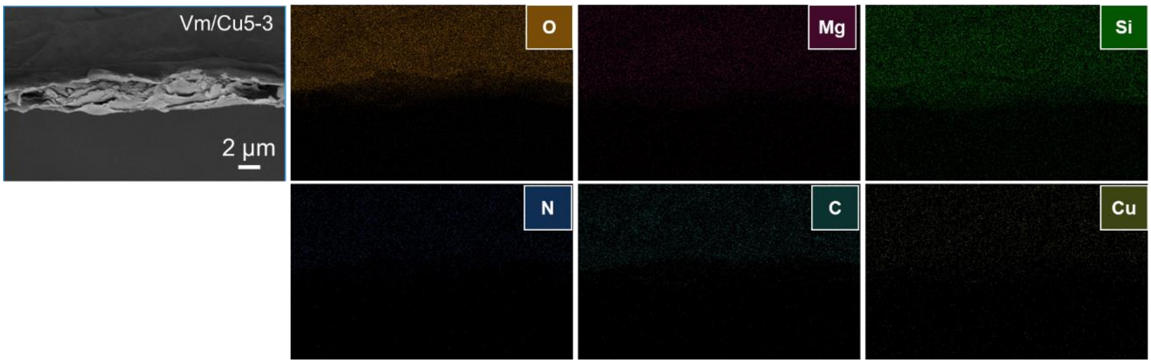

Figure S24. EDX mapping images of cross-section of Vm/Cu5-3 membrane.

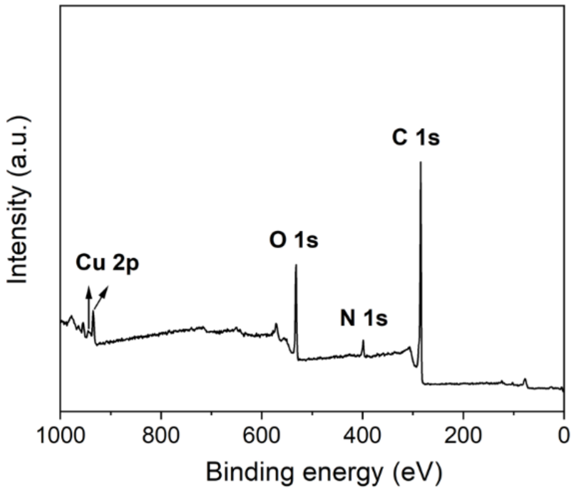

Figure S25. Full-scan XPS spectrum of CuTCPP nanosheets.

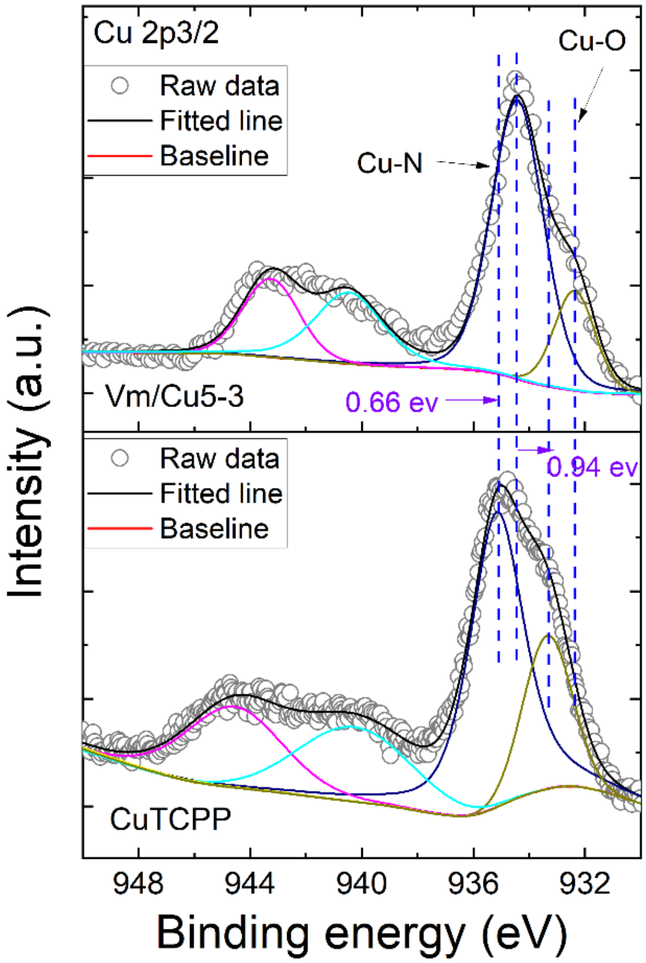

Figure S26. Cu 2p3/2 curves of Vm/Cu5-3 membrane and CuTCPP nanosheets.

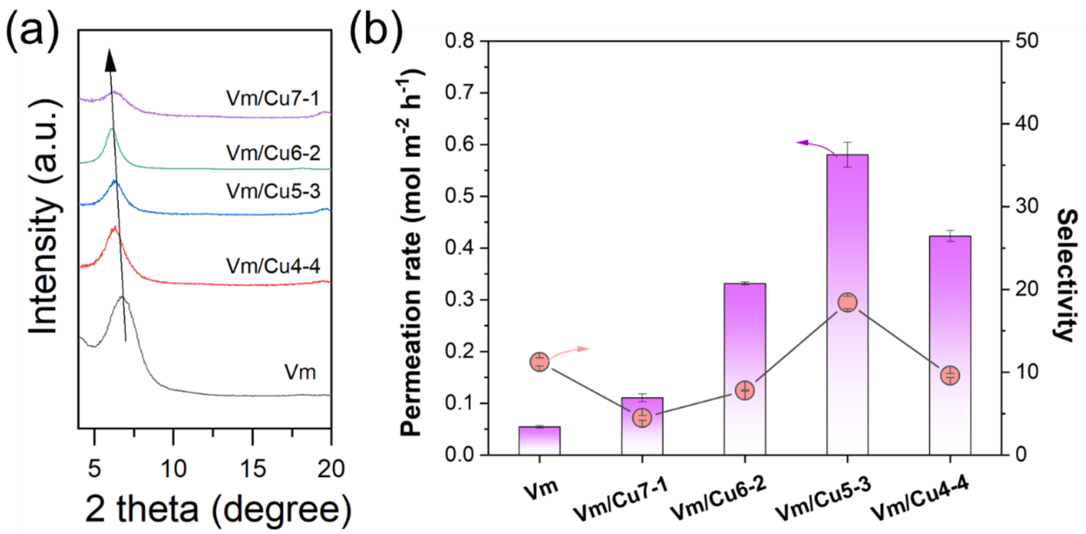

Figure S27. (a) XRD patterns and (b) ion separation performance of Vm/Cu membranes with different assembly ratio.

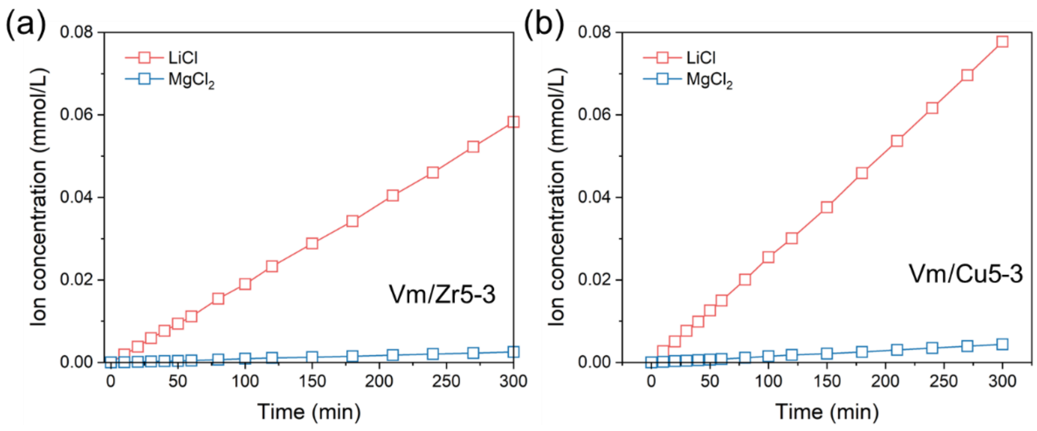

Figure S28. The ion permeation concentration in permeate side with the test time of (a) Vm/Zr5-3 and (b) Vm/Cu5-3 membrane.

The ion concentration in the permeate side increases linearly with time over the whole testing period, indicating that a steady-state permeation process is rapidly established and maintained during the measurements. Based on the linear fitting of concentration-time curves, the ion permeation rate was calculated by normalizing the amount of permeated ions with respect to the effective membrane area and the testing duration. Notably, the effective membrane area used for flux calculation was determined from optical images using ImageJ software, ensuring an accurate evaluation of the active permeation area.

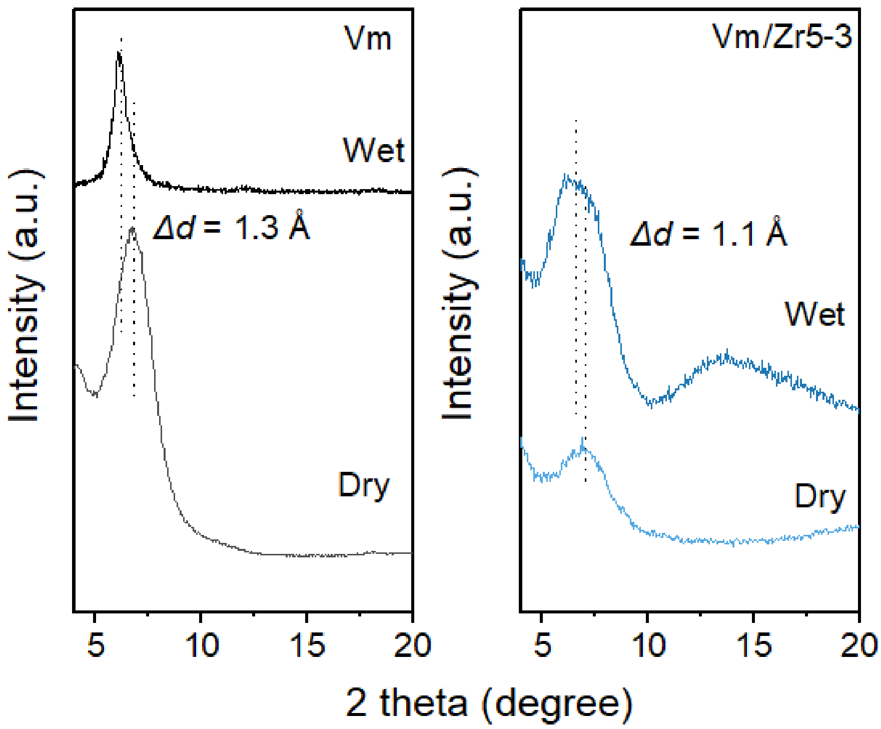

Figure S29. XRD patterns of Vm and Vm/Zr5-3 membrane in dry and wet condition.

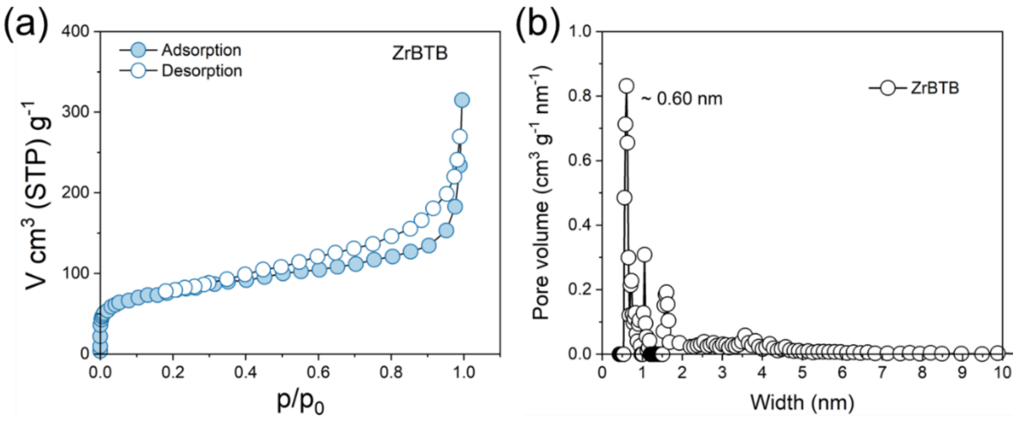

Figure S30. (a) Nitrogen adsorption-desorption curve of ZrBTB and (b) corresponding pore size distribution.

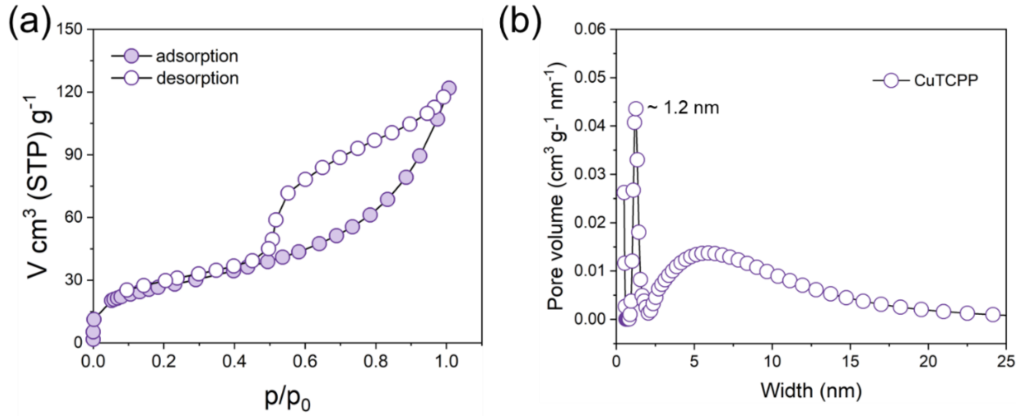

Figure S31. (a) Nitrogen adsorption-desorption curve of CuTCPP and (b) corresponding pore size distribution.

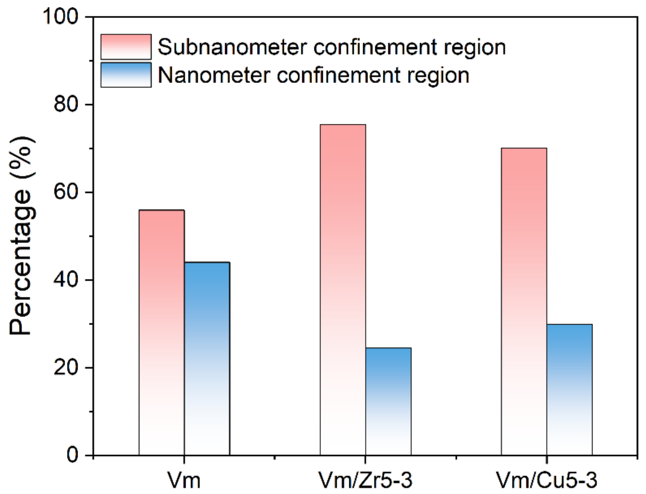

Figure S32. Percentage of sub-nanometer confinement region and nanometer confinement region for Vm, Vm/Zr5-3 and Vm/Cu5-3.

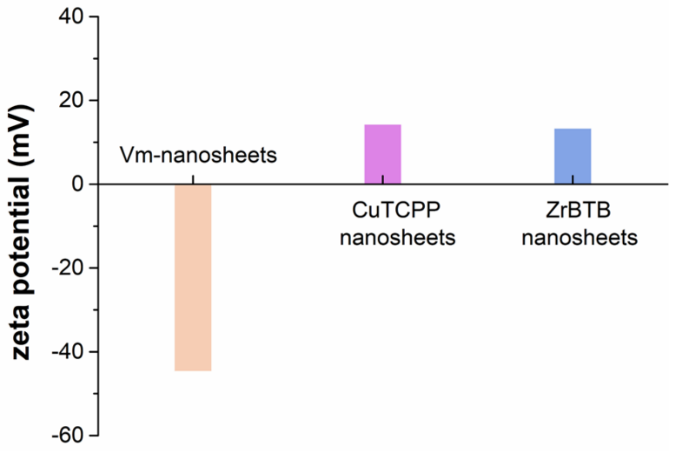

Figure S33. Zeta potential of Vm, CuTCPP and ZrBTB nanosheets dispersion.

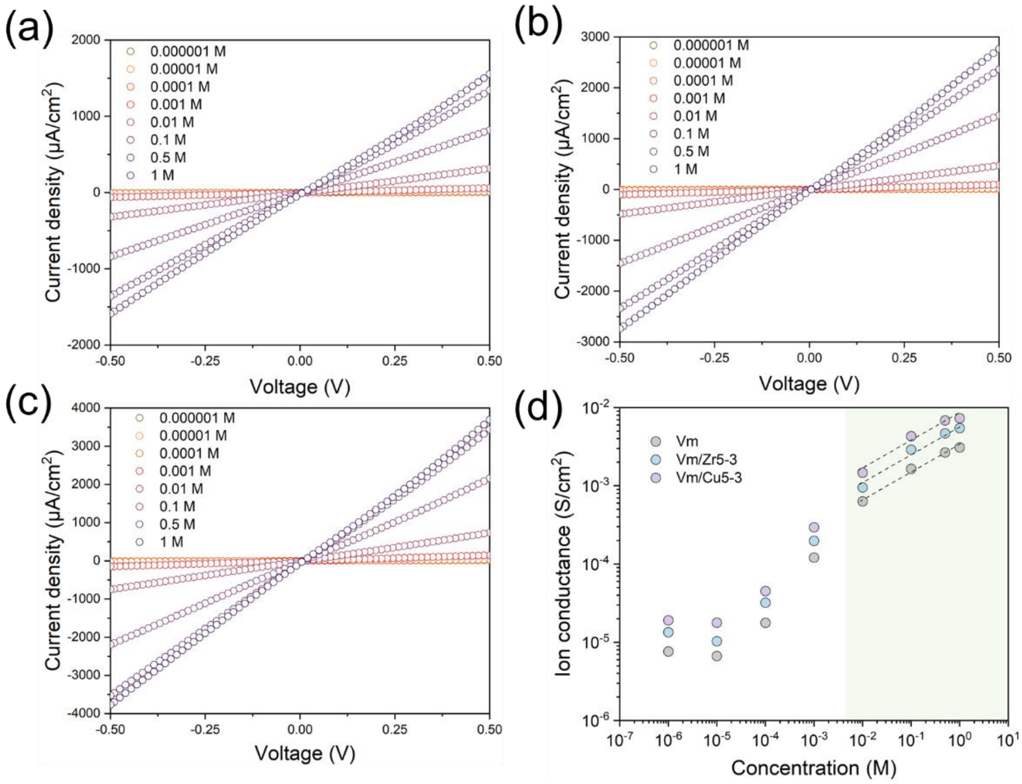

Figure S34. Current-voltage curves of (a) Vm, (b) Vm/Zr5-3, (c) Vm/Cu5-3 membrane under different concentration LiCl solution and (d) Ion conductance-concentration plots through Vm, Vm/Zr5-3 and Vm/Cu5-3 membranes.

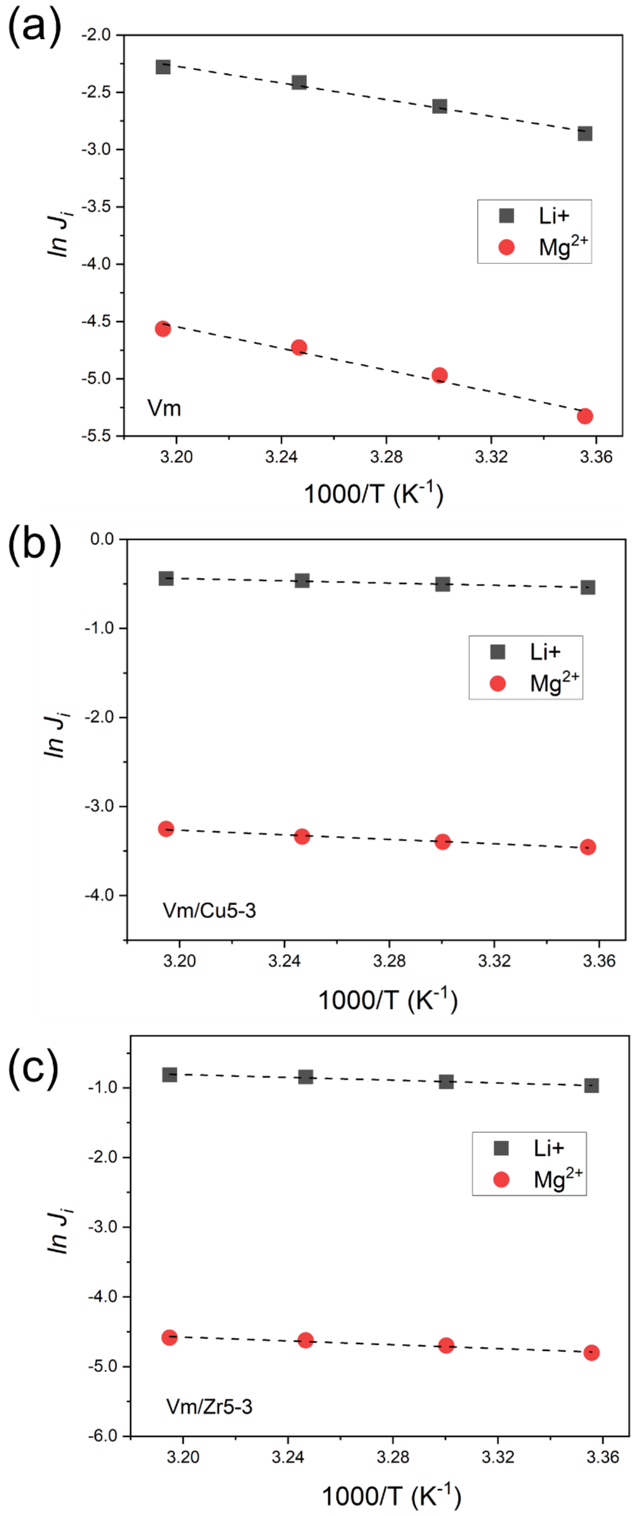

Figure S35. Ion permeation rate change plots with the increasing temperature of (a) Vm, (b) Vm/Cu5-3 and (c) Vm/Zr5-3 membrane, respectively.

Table S1. Comparison of Li^{+}/Mg^{2+} separation performance based on diffusion separation process for different types of membranes.

Membrane	Feed concentration	Li^{+} permeation rate (mol m^{-2} h^{-1})	Selectivity	References
GONS2-6	0.2 M	0.092	Li/Mg = 10.7	[14]
JGOM	0.2 M	0.24	Li/Mg = 43.5	[15]
nGOM	0.2 M	0.35	Li/Mg = 8.3	
GOM	0.2 M	0.26	Li/Mg = 1.7	
MXene	0.2 M	1.4	Li/Mg =8.8	[16]
MLM-EDTA	0.2 M	0.02	Li/Mg =28.5	[17]
EtOH-MXene	0.2 M	0.012	Li/Mg =2	[18]
MXene/PSS	0.2 M	0.08	Li/Mg =28	[19]
M-10	0.2 M	~0.14	Li/Mg =29.3	[20]
PP-MM	0.2 M	~0.05	Li/Mg =22.8	[21]
MXene/CNT	0.1 M	0.0491	Li/Mg =54.5	[22]
VmNS2-6	0.2 M	0.135	Li/Mg = 30.9	[14]
HSO3-UiO-66@QPPO-20%	1 M	0.238	Li/Mg = 5.92	[23]
UiO-66	0.1 M	0.31	Li/Mg = 8.6	[24]
DB15C5@ UiO-66	0.1 M	0.32	Li/Mg = 9.3	
DB18C6@ UiO-66	0.1 M	0.32	Li/Mg = 13	
UiO-66@NTDS	0.1 M	0.34	Li/Mg = 30	[25]
UiO-66	0.1 M	0.29	Li/Mg = 5	
Vm-0.6 μm	0.2 M	0.126	Li/Mg = 1.64	This work
Vm-1.8 μm	0.2 M	0.0546	Li/Mg = 11.24	
Vm-3.2 μm	0.2 M	0.0105	Li/Mg = 16.24	
Vm/Zr7-1	0.2 M	0.0846	Li/Mg = 18.3	
Vm/Zr6-2	0.2 M	0.137	Li/Mg = 25.61	
Vm/Zr5-3	0.2 M	0.379	Li/Mg = 46.23	

Vm/Zr4-4	0.2 M	0.398	Li/Mg = 19.84	
Vm/Cu7-1	0.2 M	0.111	Li/Mg = 4.48	
Vm/Cu 6-2	0.2 M	0.331	Li/Mg = 7.78	
Vm/Cu 5-3	0.2 M	0.58	Li/Mg = 18.4	
Vm/Cu 4-4	0.2 M	0.423	Li/Mg = 9.6	

Table S2. Summarization of relaxation time at different confinement region for Vm, Vm/Zr and Vm/Cu membranes

Membrane	I: subnanometer confinement region		II: nanometer confinement region
	$Peak_1$	$Peak_1'$	$Peak_2$
Vm	0.157 ms	2.69 ms	67.44 ms
Vm/Zr5-3	0.183 ms	2.31 ms	19.38 ms
Vm/Cu5-3	0.227 ms	2.95 ms	34.9 ms

References:

1. Pang, S.; Dai, L.; Yi, Z.; Qu, K.; Wang, Y.; Wu, Y.; Fang, C.; Huang, K.; Xu, Z., 2D nanofluidic vermiculite membranes with self-confinement channels and recognition sites for ultrafast lithium ion-selective transport. *Journal of Membrane Science* **2023,** *687*, 122054. DOI https://doi.org/10.1016/j.memsci.2023.122054.
2. Yuan, H.; Li, K.; Shi, D.; Yang, H.; Yu, X.; Fan, W.; Buenconsejo, P. J. S.; Zhao, D., Large-Area Fabrication of Ultrathin Metal-Organic Framework Membranes. *Advanced Materials* **2023,** *35* (18), 2211859. DOI https://doi.org/10.1002/adma.202211859.
3. Lu, J.; Yoshida, Y.; Maesato, M.; Kitagawa, H., High-Performance All-Solid-State Proton Rectifier Using a Heterogeneous Membrane Composed of Coordination Polymer and Layered Double Hydroxide. *Angewandte Chemie International Edition* **2022,** *61* (50), e202213077. DOI https://doi.org/10.1002/anie.202213077.
4. Rappé, A. K.; Casewit, C. J.; Colwell, K.; Goddard III, W. A.; Skiff, W. M., UFF, a full periodic table force field for molecular mechanics and molecular dynamics simulations. *Journal of the American chemical society* **1992,** *114* (25), 10024-10035.
5. Nosé, S., A unified formulation of the constant temperature molecular dynamics methods. *The Journal of chemical physics* **1984,** *81* (1), 511-519.
6. Neese, F., Software update: the ORCA program system, version 4.0. *Wiley Interdisciplinary Reviews: Computational Molecular Science* **2018,** *8* (1), e1327.
7. Grimme, S., Density functional theory with London dispersion corrections. *Wiley Interdisciplinary Reviews: Computational Molecular Science* **2011,** *1* (2), 211-228.
8. Perdew, J. P.; Burke, K.; Ernzerhof, M., Generalized gradient approximation made simple. *Physical review letters* **1996,** *77* (18), 3865.
9. Neese, F., The ORCA program system. *Wiley Interdisciplinary Reviews: Computational Molecular Science* **2012,** *2* (1), 73-78.
10. Grimme, S.; Ehrlich, S.; Goerigk, L., Effect of the damping function in dispersion corrected density functional theory. *Journal of computational chemistry* **2011,** *32* (7), 1456-1465.
11. Lu, T.; Chen, F., Multiwfn: A multifunctional wavefunction analyzer. *Journal of computational chemistry* **2012,** *33* (5), 580-592.
12. Lu, T., A comprehensive electron wavefunction analysis toolbox for chemists, Multiwfn. *The Journal of chemical physics* **2024,** *161* (8).
13. Humphrey, W.; Dalke, A.; Schulten, K., VMD: visual molecular dynamics. *Journal of molecular graphics* **1996,** *14* (1), 33-38.
14. Dai, L.; Pang, S.; Li, S.; Yi, Z.; Qu, K.; Wang, Y.; Wu, Y.; Li, S.; Lei, L.; Huang, K.; Guo, X.; Xu, Z., Freestanding two-dimensional nanofluidic membranes modulated by zwitterionic polyelectrolyte for mono-/di-valent ions selectivity transport. *Journal of Membrane Science* **2023,** *677*, 121621. DOI https://doi.org/10.1016/j.memsci.2023.121621.
15. Wang, S.; Fang, C.; Huang, Y.; Yi, R.; Wu, M.; Wang, Y.; Li, F.; Zhu, L.; Liang, S.; Chen, L., Bio-Inspired 2D Asymmetric Nanochannels for High-Resolution Li+/Mg2+ Separation. *Angewandte Chemie International Edition* **2025,** *n/a* (n/a), e202512310. DOI https://doi.org/10.1002/anie.202512310.

16. Ren, C. E.; Hatzell, K. B.; Alhabeb, M.; Ling, Z.; Mahmoud, K. A.; Gogotsi, Y., Charge- and Size-Selective Ion Sieving Through Ti3C2Tx MXene Membranes. *The Journal of Physical Chemistry Letters* **2015,** *6* (20), 4026-4031. DOI 10.1021/acs.jpclett.5b01895.
17. Xu, R.; Kang, Y.; Zhang, W.; Pan, B.; Zhang, X., Two-dimensional MXene membranes with biomimetic sub-nanochannels for enhanced cation sieving. *Nature Communications* **2023,** *14* (1), 4907. DOI 10.1038/s41467-023-40742-8.
18. Kang, Y.; Hu, T.; Wang, Y.; He, K.; Wang, Z.; Hora, Y.; Zhao, W.; Xu, R.; Chen, Y.; Xie, Z.; Wang, H.; Gu, Q.; Zhang, X., Nanoconfinement enabled non-covalently decorated MXene membranes for ion-sieving. *Nature Communications* **2023,** *14* (1), 4075. DOI 10.1038/s41467-023-39533-y.
19. Lu, Z.; Wu, Y.; Ding, L.; Wei, Y.; Wang, H., A Lamellar MXene (Ti3C2Tx)/PSS Composite Membrane for Fast and Selective Lithium-Ion Separation. *Angewandte Chemie International Edition* **2021,** *60* (41), 22265-22269. DOI https://doi.org/10.1002/anie.202108801.
20. Wu, Y.; Jia, M.; Li, S.; Lu, Z.; Wei, Y.; Wang, H., Positively charged Ti3C2Tx membrane for enhanced Li+/Mg2+ sieving. *Journal of Membrane Science* **2025,** *717*, 123604. DOI https://doi.org/10.1016/j.memsci.2024.123604.
21. Wang, X.; Xing, J.; Wei, G.; Chen, S.; Quan, X., Pyrrole functionalized Ti3C2Tx MXene membrane with sub-nanochannels for selective mono/divalent ions separation. *Journal of Water Process Engineering* **2025,** *73*, 107671. DOI https://doi.org/10.1016/j.jwpe.2025.107671.
22. Lu, J.; Dai, C.; Li, S.; Zou, D.; Sun, Y.; Jing, W., Ultraefficient Li+/Mg2+ separation with MXene/CNT membranes under electric field assistance. *Separation and Purification Technology* **2024,** *338*, 126508. DOI https://doi.org/10.1016/j.seppur.2024.126508.
23. Zeng, X.; Xu, L.; Deng, T.; Wang, Y.; Xu, W.; Zhang, W., Anionic MOFs Embedded in Anion-Exchange Membranes for the Separation of Lithium/Magnesium Cations. *ACS Sustainable Chemistry & Engineering* **2023,** *11* (35), 12877-12887. DOI 10.1021/acssuschemeng.3c00891.
24. Xu, T.; Wu, B.; Li, W.; Li, Y.; Zhu, Y.; Sheng, F.; Li, Q.; Ge, L.; Li, X.; Wang, H.; Xu, T., Perfect confinement of crown ethers in MOF membrane for complete dehydration and fast transport of monovalent ions. *Science Advances* **2024,** *10* (19), eadn0944. DOI 10.1126/sciadv.adn0944.
25. Li, W.; Xu, T.; Sheng, F.; Wang, Y.; Li, Y.; Xia, Y.; Wu, B.; Li, X.; Xu, T., UiO-66 membranes with confined naphthalene disulfonic acid for selective monovalent ion separation. *Journal of Membrane Science* **2024,** *703*, 122829. DOI https://doi.org/10.1016/j.memsci.2024.122829.
